# Pangenome-based structural variant imputation enables large-scale genotype-phenotype studies in dairy cattle

**DOI:** 10.1038/s41467-026-75219-x

**Published:** 2026-07-03

**Authors:** Liu Yang, Junjian Wang, Kristen Kuhn, Wenli Li, Geoffrey Zanton, Mahesh Neupane, Clarissa Boschiero, John B. Cole, Bingjie Li, Congjun Li, Ransom L. Baldwin, VI, Curtis P. Van Tassell, Benjamin D. Rosen, Timothy P. L. Smith, Jicai Jiang, Lingzhao Fang, Li Ma, George E. Liu

**Affiliations:** 1 Animal Genomics and Improvement Laboratory, Beltsville Agricultural Research Center, Agricultural Research Service, United States Department of Agriculture, Beltsville, MD USA; 2 Department of Animal and Avian Sciences, University of Maryland, College Park, MD USA; 3 Department of Animal Science, North Carolina State University, Raleigh, NC USA; 4 USDA, ARS, U.S. Meat Animal Research Center (USMARC), Clay Center, NE USA; 5 US Dairy Forage Research Center, USDA-ARS, Madison, WI USA; 6 Council on Dairy Cattle Breeding, Bowie, MD USA; 7 Department of Animal Sciences, Donald Henry Barron Reproductive and Perinatal Biology Research Program, and the Genetics Institute, University of Florida, Gainesville, FL USA; 8 Centre for Genetic Improvement of Livestock, University of Guelph, Guelph, ON Canada; 9 Department of Animal and Veterinary Sciences, Scotland’s Rural College, Midlothian, UK; 10 Quantitative Genetics and Genomics (QGG), Aarhus University, Aarhus, Denmark

**Keywords:** Agricultural genetics, Structural variation, Genomics

## Abstract

Pangenomes of several species have been assembled recently, facilitating the detection and genotyping of structural variants. As part of the FarmGTEx Project, we previously constructed a Holstein pangenome (H20D) based on 40 phased haploid assemblies. Here, we use this breed specific pangenome to genotype 93,059 structural variants from whole-genome sequences of 1,571 cattle. We then develop a Holstein pangenome variation imputation reference panel we name HolPIP. Leveraging HolPIP, we impute 86.65% (68,354/78,886) of structural variants for 50,299 bulls with Beagle R² ≥ 0.8. Using these imputed structural variants and phenotypes for 43 complex traits, we conduct GWAS, identifying 1,225 structural variant-trait associations. We next use fine-mapping to prioritize 32 high-confidence candidate structural variants, including a 75-bp deletion in *ANKRD11* linked to dairy form, rump width, and stature, as well as an insertion in *DHX32* associated with RNA metabolism. Compared to SNPs across various functional annotations, structural variants show a stronger genome-wide enrichment across most complex traits in cattle, suggesting that structural variants may have an important contribution to the genetic basis of dairy traits.

## Abstract (web summary)

The FarmGTEx project previously developed a Holstein cattle pangenome. Here, the authors use that pangenome to explore structural variation and imputation in Holstein cattle, and use these data to perform a GWAS for economically important traits.

## Introduction

Understanding genetic diversity is essential for progress in both human health and livestock productivity^1–4^. While single-nucleotide polymorphisms (SNPs) have traditionally been the focus of genomic studies, structural variants (SVs), including insertions (INS), deletions (DEL), and others, also contribute significantly to genetic and phenotypic diversity but have remained underrepresented due to technical challenges in their detection, genotyping, and imputation^5–10^. Long-read sequencing and pangenome frameworks greatly improve the detection of complex SVs, especially in repetitive regions. Because a single reference cannot capture full SV diversity, pangenomes built from multiple assemblies provide a broader representation^11^. Pangenome graphs model shared and alternative haplotypes as nodes and paths, enabling more accurate SV detection. Tools such as PanGenie^12^ and Giraffe (from the *vg* toolkit)^13^ use these graphs to efficiently genotype SVs from short-read data informed by long-read variant catalogs^14^.

Recent advances have demonstrated that SV and pangenome-based genotyping are broadly useful across species. In humans, a recent long-read sequencing panel of 888 individuals from the 1000 Genomes Project was used to impute SVs into ~488,000 participants from the UK Biobank and perform a proof-of-concept SV-GWAS, uncovering thousands of significant SV associations and highlighting the value of SVs for fine-mapping and causal gene prioritization^15,16^. In plants, SVs identified via graph- or pan-genome approaches often underlie agronomically important traits, with SV-based GWAS revealing loci for fruit flavor and yield in tomato^17,18^ and trait-associated insertions affecting grain number or stress-response in rice and millet species^19,20^.

Pangenomic studies in cattle and sheep have improved SV detection, cross-breed comparisons, and trait mapping, highlighting the genetic basis of adaptation, productivity, and resilience^21–28^. However, these studies are limited in sample size and integration with large-scale GWAS. Holsteins, a major global dairy breed, have mostly been analyzed using SNP chips, which miss many SVs affecting complex traits^29,30^. Long-read Holstein pangenomes now reveal breed-level SV diversity^31^, but pangenome-based SV imputation has not been systematically evaluated, leaving a gap in applying SVs to trait mapping and genomic selection.

As part of the FarmGTEx-cattle Phase 1 Project^32^, we previously constructed a Holstein-specific pangenome (H20D) using 40 phased haploid assemblies from 20 cattle^33^. In this study, we used a combination of the Holstein-specific pangenome and graph-based genotyping approaches to comprehensively characterize both single-nucleotide and structural variation from short-read sequencing data in a large cattle population. We then developed a pangenome-based reference panel to enable genome-wide imputation of SVs at a population scale. By integrating these data with phenotypes for economically important traits, our subsequent genome-wide analyses identified multiple SV associated with variation in growth, production, and other complex traits. Together, these results demonstrate the power of breed-specific pangenomes and SV imputation for capturing previously underexplored genetic variation and indicate their potential to enhance genomic selection and accelerate genetic improvement in cattle.

## Results

### Pangenome-based SV genotyping in 1571 whole-genome sequencing samples

**Fig. 1:**
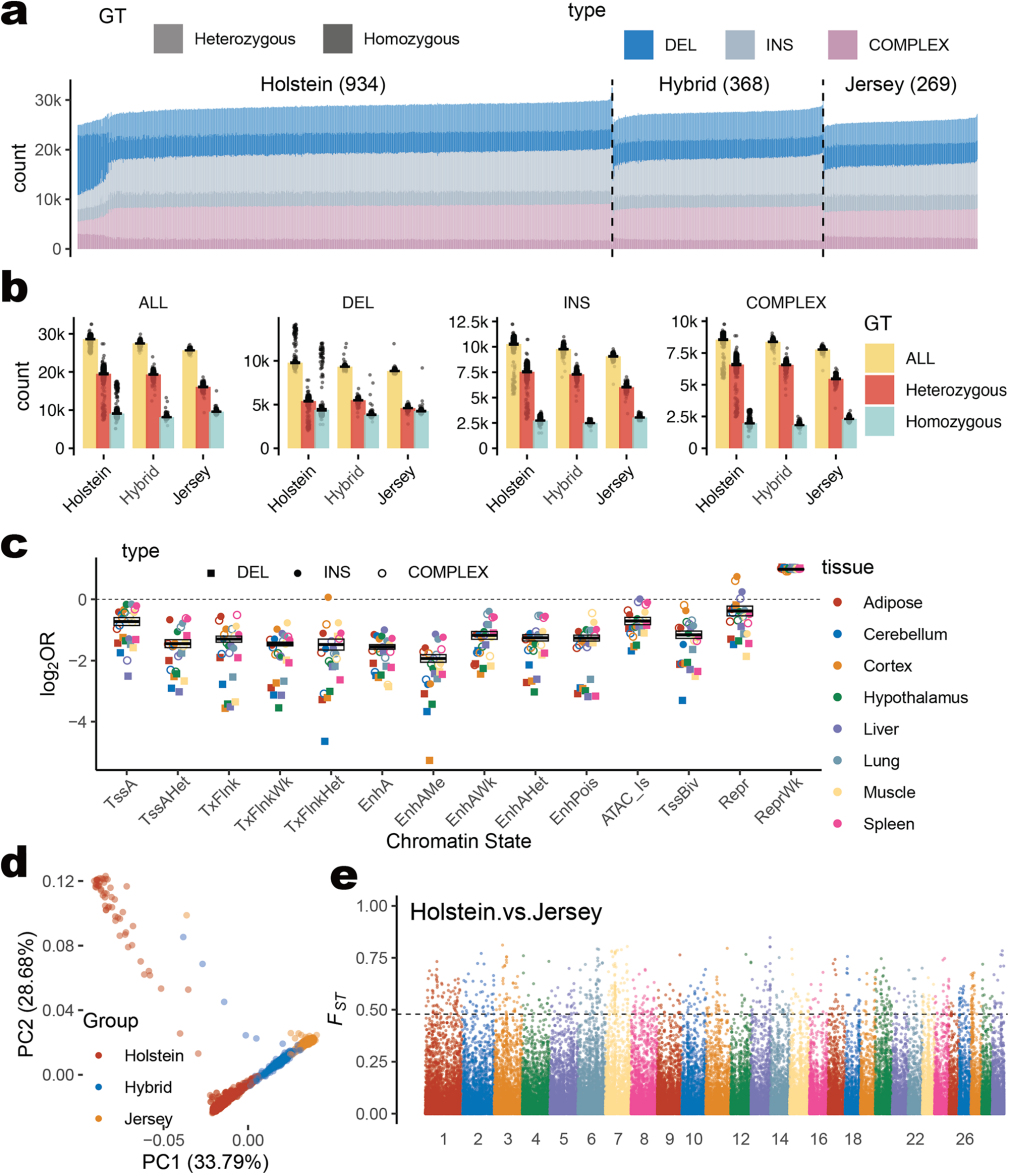
Pangenome-based genotyping of SVs from 1571 cattle. **a** Number of SV across 1571 cattle. The k in the y-axis denotes one thousand. **b** Heterozygous and homozygous SV counts per individual across Holstein (*n* = 934), Jersey (*n* = 269), and Holstein–Jersey hybrid cattle (*n* = 368). Each data point represents one independent animal (biological replicate). Bars represent the mean, and error bars indicate the 95% confidence interval assuming a normal distribution. No technical replicates were included. **c** Overlap between 93,059 SVs (19,770 DEL, 37,684 INS, and 35,605 COMPLEX events) and 14 chromatin states across eight cattle tissues comprising 3,000,212 annotated chromatin-state regions. The y-axis denotes the overlapped length transformed by log_10_. The k and M denote thousand and million, respectively. Different colors denote each tissue. Bars represent the sample mean, lower, and upper Gaussian 95% confidence limits for each individual, based on the t-distribution. The y-axis denotes the odds ratio (OR) for the length of each repeat overlapped with SVs, using the genome total length as background, which is transformed by log_2_. The dashed line at *y* = 0 indicates that values above the line show a positive association with SVs and repeats, whereas values below this line indicate a negative association. A total of 14 chromatin states include Strongly active promoters/transcripts (TssA), Flanking active TSS without ATAC (TssAHet), Transcribed at gene (TxFlnk), Weak transcribed at gene (TxFlnkWk), Transcribed region without ATAC (TxFlnkHet), Strong enhancer (EnhA), Medium enhancer with ATAC (EnhAMe), Weak active enhancer (EnhAWk), Active enhancer no ATAC (EnhAHet), Poised enhancer (EnhPois), ATAC island (ATAC_Is), Bivalent/poised TSS (TssBiv), Repressed polycomb (Repr), and Weak repressed polycomb (ReprWk). **d** Principal component analysis (PCA) of 1571 cattle based on PanGenie SV with MAF ≥ 0.05. The explained variation percentages for parentheses for PC1 and PC2 (in parentheses) were calculated by PLINK2. **e** Manhattan plot showing *F*_*ST*_ statistics of SVs for Holstein vs. Jersey. SVs above the top 1% threshold lines (black) were considered as candidate selections.

We previously generated a Holstein pangenome using 40 haplotype-resolution assemblies from 20 HiFi samples^33^. Using the phased pangenome SV reference, we genotyped 934 Holsteins, 269 Jerseys, and 368 Holstein-Jersey Hybrids (total: 1571, Supplementary Data 1) via PanGenie^34^, detecting 12.58 M autosomal SNVs and 93,059 SVs (19,770 DEL, 37,684 INS, 35,605 COMPLEX) (Fig. 1a, Supplementary Fig. 1a, and Supplementary Data 2). “COMPLEX” refers to variant alleles located within pangenome-graph bubbles containing more than two branches, representing sequence changes of at least two base pairs that cannot be classified simply as DEL or INS. The non-redundant SV length of 56.95 Mb (DEL: 18.34 Mb, INS: 25.29 Mb, and COMPLEX: 29.04 Mb) corresponded to 2.31% of the ARS-UCD v2.0 genome (DEL: 0.7%, INS: 1.0%, and COMPLEX: 1.17%). Holsteins had the most SVs per sample (28,825), followed by hybrids (27,497) and Jerseys (25,656). Holsteins had 4,011 more heterozygous SVs than Jerseys, while Jerseys had 870 more homozygous SVs than Holsteins. Similarly, Hybrids had 3,387 more heterozygous SVs than Jersey, while Jersey had 1532 more homozygous SVs than Hybrids (Fig. 1a, b, Supplementary Data 3 and Supplementary Data 4). Among all SVs across the 1571 samples, 76,284 (81.97%) were classified as common (5% ≤ frequency ≤95%), with breakdowns by type: 17,171 for DEL, 30,630 for INS, and 28,483 for COMPLEX events. DEL events had the highest major-allele proportion ( ≥ 95% frequency) at 8.27%, compared to 1.13% for INS and 0.72% for COMPLEX events (Supplementary Fig. 1b, Supplementary Data 5, and Supplementary Data 6).

### Annotation of the detected SV regions from 1571 WGS samples

Most insertion SVs overlapped repetitive elements (79.19%, 33.48 Mb), particularly long interspersed nuclear elements (LINEs, 65.45%, 21.91 Mb), short interspersed nuclear elements (SINEs, 17.34%, 5.81 Mb), and long terminal repeats (LTRs, 11.79%, 3.95 Mb) (Supplementary Fig. 1c and Supplementary Data 7). The overlap proportions were largely consistent across SV types, although satellite sequences had the highest odds ratio (OR = 3.0), while DNA transposons and SINEs had lower overlap probabilities (Supplementary Fig. 1d and Supplementary Data 7).

We further annotated 40,801 (43.84%) SVs overlapping 7967 genes, including 6107 (76.65%) protein-coding genes. More specifically, 4755 genes were mapped to 8742 DELs, 5029 genes to 16,200 INSs, and 3518 genes to 15,859 COMPLEXs (Supplementary Data 8). Among gene biotypes overlapping SVs, Immunoglobulin C region, misc_RNA, Immunoglobulin V segment, rRNA, snoRNA, snRNA, ncRNA, and pseudogene had the highest odds ratios (OR) (near or over 2) (Supplementary Data 9). Conversely, miRNAs had the lowest SV overlap. Additionally, most SVs were in intergenic regions rather than genic regions, especially outside exons and coding sequences (CDS), aligning with evolutionary selection pressures (Supplementary Fig. 1e and Supplementary Data 10).

We annotated those 19,770 DEL, 37,684 INS, and 35,605 COMPLEX events across 3,000,212 regions of 14 chromatin states in 8 cattle tissues, including adipose, cerebellum, cortex, hypothalamus, liver, lung, muscle, and spleen. Enhancers and promoters generally exhibited low association with SVs (OR < 1), with the weakest overlap in the EnhAMe regions (medium enhancers with ATAC, average OR = 0.331 across tissues) (Fig. 1c and Supplementary Data 11). The enrichment of SVs in the ReprWk (Weak repressed polycomb) regions may suggest that SVs are likely to localize in low-functional areas^35^.

Quantitative trait locus (QTL) analysis can highlight functional associations between SVs and complex traits^36^. We examined 163,443 QTLs (after filtering for length <1 Mb) across 510 different cattle traits from Animal QTLdb (release 54)^37^. Of these, 2276 QTLs (1.39%) associated with 29.0% (148/510) of traits overlapped with SVs, with significant enrichment (OR ≥ 2) for 27 traits, including water holding capacity (OR = 44.71), endometritis (22.36), milk cis-vaccenic acid content (22.36), young stock survival (19.87), and calving index (11.77) (Supplementary Fig. 1f and Supplementary Data 12).

### Population stratification by pangenome SVs

Principal component analysis (PCA) of PanGenie SNPs/SVs (with minor allele frequency—MAF ≥ 0.05) revealed three distinct clusters among the 1571 samples, corresponding to the breed groups: 934 Holstein, 269 Jersey, and 368 Hybrid samples (Fig. 1d and Supplementary Fig. 2a). PCA of PanGenie SNPs/SVs exhibited a similar pattern to that of DeepVariant SNPs (Supplementary Fig. 2b). We used SV genotypes to identify selection signatures and estimated fixation index (*F*_*ST*_) statistics to detect SV frequency differences among the Holstein, Jersey, and Hybrid groups. We calculated SV-based *F*_*ST*_ and identified lineage-differential SVs as those with the highest *F*_*ST*_ (top 1%) between Holstein, Jersey, and Hybrid cattle (Fig. 1e, Supplementary Fig. 1c, and Supplementary Data 13). A total of 65 high-*F*_*ST*_ SVs were shared with Holstein vs. Jersey (0.479), Holstein vs. Hybrid (0.253), and Jersey vs. Hybrid (0.200) comparison pairs. They include 22 DEL, 20 INS, and 23 COMPLEX events with a nonredundant length of 77.7 kb and overlapping with 16 genes, including *LPP*, *PARD3B*, *CENPC*, *SULT1D1*, *DDX46*, *LOC100848799*, *JAKMIP2*, *SKIC3*, *RNF38*, *PRKN*, *EFCAB11*, *DOK5*, *CNTN5*, *CDH13*, *EMB*, and *LDLRAD4* (Supplementary Data 14). Notably, *SULT1D1* was affected by both a DEL (6:85,266,136-85,267,259) and a COMPLEX SV (6:85,271,275-85,271,783), while an INS (7:46,280,720-46,280,825) within *DDX46* was located in its CDS, with potential functional relevance in body height^38^.

### Pangenome enables SV imputation from SNPs

Based on the pangenome variations across 1571 cattle genotyped by PanGenie with the phased single-breed pangenome (H20D), we tested the imputation accuracy of Holstein-related pangenome SV across a randomly selected sample of 750 Holsteins using a 10× cross-validation approach, in which the 750 Holsteins were randomly divided into ten equal subsets and imputation accuracy was evaluated by iteratively training on nine subsets and testing on the remaining one. One-tenth of the samples were randomly selected as the test set, with 10%, 20%, and 50% of their genotypes masked as missing. The remaining samples were used to train the imputation model, which was self-phased as the reference panel. Using RTG Tools, we compared genotypes before masking with those after imputation for each test sample. We first tested SVs on each cattle chromosome to optimize computational resources. Using Beagle (v5.4), we observed consistent imputation accuracy across chromosomes, with an average F1 score of 0.898, ranging from 0.880 (chr25) to 0.906 (chr1) (Supplementary Fig. 3a and Supplementary Data 15). We then compared the imputation performance of Beagle and Minimac (v4.1.6) using SVs and SNPs, respectively. On average, Beagle outperformed Minimac, achieving higher F1 scores, precision, and sensitivity in our analysis by 6.86%, 4.00%, and 9.31%, respectively (Supplementary Fig. 3b and Supplementary Data 16). We then selected Beagle and chr10 as an example to further explore the factors influencing SV imputation.

### Using SV to impute SVs

When using SV to impute SVs (size ≥ 50 bp), F1 scores decreased with increasing masking, from 0.935 (10%) to 0.889 (50%), indicating that marker density affects imputation accuracy (Supplementary Fig. 4a and Supplementary Data 17). Among SV types, DELs were most accurately imputed (F1: 0.95 at 10%, 0.94 at 50%), followed by COMPLEX (0.94, 0.88) and INS (0.92, 0.91) events (Supplementary Fig. 4b and Supplementary Data 17). This trend suggests that COMPLEX SV imputation performance depends more on marker frequency, suggesting that they may serve as more sensitive indicators of marker availability and distribution (Supplementary Fig. 4b and Supplementary Data 17). Among the genotype categories, homozygous SVs (HOM: 1/1) achieved the highest F1 score (0.93) when 50% of the SVs were masked, surpassing heterozygous SVs (HET: 0/1 or 1/0, 0.84), all SVs combined (ALT: 1/1, 0/1, or 1/0, 0.89), and non-SVs (REF: 0/0, 0.87) (Supplementary Fig. 4c and Supplementary Data 18).

### Using SNPs to impute SVs

**Fig. 2:**
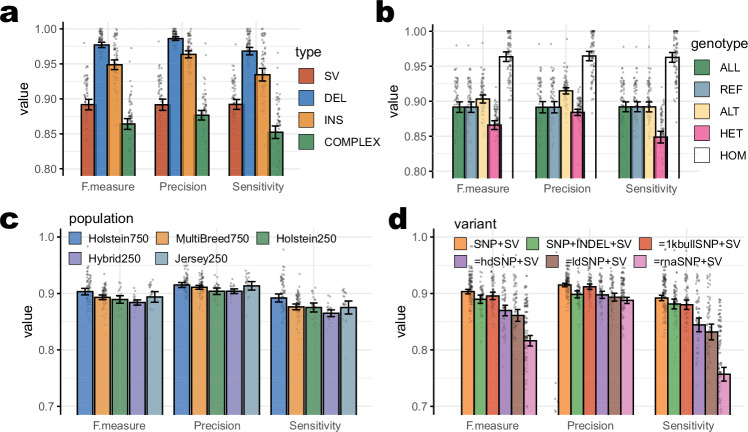
Consistency of imputing SVs by SNPs. **a** Imputation performance for different SV genotypes using SNP-based imputation, where SNPs were used to impute SVs with two combined marker datasets (SVs + SNPs). All SVs were masked as missing for test samples (*n* = 75). Bars represent the mean, and error bars indicate the 95% confidence interval assuming a normal distribution. **b** Imputation performance for different SV genotypes using SNPs for test samples (*n* = 75). Bars represent the mean, and error bars indicate the 95% confidence interval assuming a normal distribution. **c** Imputation performance of SNP-based SV imputation with varying sample sizes and population compositions. The 750 Multibreed dataset includes 250 Holstein (*n* = 75), 250 Hybrid (*n* = 75), and 250 Jersey (*n* = 75) samples. Bars represent the mean, and error bars indicate the 95% confidence interval assuming a normal distribution. **d** Imputation performance of SNP-based SV imputation for different variant types in tested samples (*n* = 75). “SNP + SV” denotes combined SNP and SV for reference samples, with only SNPs available for test samples. “SNP + INDEL + SV” includes SNPs, INDELs, and SVs in the reference set, while test samples have only SNPs and INDELs for SV imputation. =1kBullSNP refers to SNPs extracted from whole-genome sequencing (WGS) data from the 1000 Bulls Project (Run 8), similar to hdSNP (Bovine 777 K high-density SNP array), ldSNP (BovineSNP50 Chip), and rnaSNP (from 19 RNA-seq samples and intersected with CattleGTEx SNP locations). Bars represent the mean, and error bars indicate the 95% confidence interval calculated assuming a normal distribution in all panels.

To further evaluate the imputation accuracy of pangenome SVs based on SNPs (MAF ≥ 0.01), we masked all SVs in the test samples and used SNP data as input. For reference samples, we combined the SNP matrix with SV data. In total, SV imputation based on SNPs achieved an F1 score of 0.89, with both precision and sensitivity near 0.89 (Fig. 2a and Supplementary Data 19). Among SV types, DELs showed the highest F1 score (0.98), followed by INS (0.95) and COMPLEX (0.86) (Fig. 2b). When 50% of all types of variations in the combined SV and SNP matrix were masked, SNPs outperformed other SV types, achieving the highest F1 score of 0.99, surpassing DEL (0.98), INS (0.96), and COMPLEX (0.92) (Supplementary Fig. 5a and Supplementary Data 19). For allele types, like SV imputation, homozygous variants outperformed heterozygous ones (F1: 0.96 vs. 0.87) across all types of SVs (Fig. 2e and Supplementary Data 44), while alternative alleles performed similarly to reference alleles (0.90 vs. 0.89) (Supplementary Fig. 5b and Supplementary Data 20).

### Population and sample size

We also examined the effects of breed and sample size on imputation accuracy by sampling five groups from the 1571 cattle: 250 Holsteins, 250 Hybrids, 250 Jerseys, 750 Holsteins, and a mixed group of 750 samples (250 Holsteins, 250 Hybrids, and 250 Jerseys). We found that increasing Holstein samples from 250 to 750 raised the F1 score from 0.89 to 0.90, and 250 Jerseys (0.89) performed similarly to 250 Holsteins, though they slightly outperformed 250 Hybrids by 0.01 (0.88). Among 750-sample groups, Holsteins achieved a slightly higher F1 score (0.90) than the Mixed group (0.89) (Fig. 2c and Supplementary Data 21). Overall, larger sample sizes and closer genetic relationships to the reference population improved imputation accuracy. Contrary to expectations, Jersey samples did not show lower imputation accuracy with the Holstein pangenome reference (Fig. 2c). We consider this a preliminary and unvalidated test of cross-breed imputation. The high performance observed is likely driven by a high proportion of frequent, homozygous SVs in Jerseys that are shared with the Holstein reference. However, we caution that accuracy for rare or breed-specific variants is expected to be significantly lower.

### Genome region and feature

In the repetitive regions, we found that low-complexity regions (F1 score: 0.78) and simple repeats (F1 score: 0.82) exhibited the lowest F1 scores, as compared to LTR (0.87) and LINE (0.90) (Supplementary Fig. 6a and Supplementary Data 22). These lower F1 scores reflect the challenges associated with accurately genotyping SNPs or SVs in difficult repetitive regions. We also observed the lowest F1 score in coding sequences (CDS, 0.74) within genic regions, compared to other regions such as pseudogenes (0.77), exons (0.84), introns (0.90), lncRNAs (0.91), and intergenic regions (0.91) (Supplementary Fig. 6b and Supplementary Data 23). This likely reflects the strong functional constraint in CDS, which results in fewer SVs and a higher proportion of rare, conserved variants whose breakpoints occur in sequence contexts that are more difficult to align and resolve. We speculate that these biological and technical factors, rather than differences in MAF distribution, contribute to reduced genotyping performance in CDS (Supplementary Fig. 6c–e and Supplementary Data 24).

### SNP density level

We then tested imputation accuracy across varying levels of SNP density by extracting SNPs based on their genome locations from the WGS dataset of the 1000 Bulls Project (Run 8) (=1kBullSNP, 15,758,693 SNPs), the Bovine 777 K high-density SNP array (=hdSNP, 774,316 SNPs), the BovineSNP50 Chip (=ldSNP, 68,706 SNPs), and RNA SNP datasets (1,451,919 SNPs). RNA SNPs were derived from 19 of the 20 Holstein blood samples matched with HiFi data (1 sample was missing), intersecting with the CattleGTEx SNP data^39^. As SNP density decreased across 1kBull, HD, LD, and RNA levels, sensitivity declined more rapidly than precision (Fig. 2d). Imputation accuracy also decreased, but F1 scores for SVs imputed across all SNP density levels remained above 0.8. For example, despite a sensitivity of 0.76 and a precision of 0.89, RNA SNPs achieved an F1 score of 0.82, demonstrating the robustness of SNP-based SV imputation using a fully phased single-breed pangenome as reference (Supplementary Data 25). Interestingly, adding indels to the matrix only slightly lowered SV imputation accuracy from 0.90 to 0.89 (Fig. 2g), likely because indels are more difficult to genotype reliably and can disrupt haplotype phasing or marker consistency, thereby introducing additional noise into the imputation process. No significant difference was observed between WGS-level SNPs and PanGenie SNPs, both of which demonstrated high consistency (F1 = 0.90), agreeing with the results presented in the SNV consistency evaluation section. RNA SNPs showed lower accuracy for SV imputation than HD and LD SNPs, despite their higher count, suggesting that the genomic distribution or characteristics of RNA SNPs, rather than their quantity, may influence imputation accuracy.

### Holstein pangenome imputation reference panel

Given the high accuracy of SV imputation by SNPs with the phased single-breed pangenome reference, along with the advantages of the k-mer-based (mapping-free) SV genotyping method^34^, we constructed the Holstein pangenome variation imputation reference panel (HolPIP) with 1302 Holstein-related cattle, including 934 Holstein and 368 Holstein-Jersey hybrid samples. After applying a MAF filter of ≥0.01, a total of 15,727,951 pangenome variations were genotyped by PanGenie. These included 12,512,405 SNPs, 1,250,141 short deletions (sDEL, a type of short INDEL ≤ 50 bp), 1,318,751 short insertions (sINS, INDEL ≤ 50 bp), and 553,595 short complex variations (sCOMPLEX, including multi-nucleotide polymorphisms (MNPs) ≤ 50 bp). Additionally, a total of 93,059 SVs were genotyped, including 19,770 DELs (≥50 bp), 37,684 INSs (≥50 bp), and 35,605 COMPLEXs (≥50 bp).

### Testing with SNPs from PanGenie

To further evaluate the performance of the HolPIP, we used independent WGS data from 173 Holstein samples genotyped with the phased H20D reference. We masked all SVs (82,355 SVs, MAF ≥ 0.01) as missing and performed imputation using 11,934,283 PanGenie SNPs and Beagle with the HolPIP as the reference panel. We assessed imputation accuracy using the average F1 score, precision, and sensitivity across SNP densities (1kBull (10,632,332 SNPs), HD (571,555 SNPs), LD (67,356 SNPs), and RNA (1,539,272 SNPs after filtering)), and calculated Beagle DR² for each SV locus. The DR² is Dosage *R*², the squared correlation between the true genotype dosage and the imputed dosage.

**Fig. 3:**
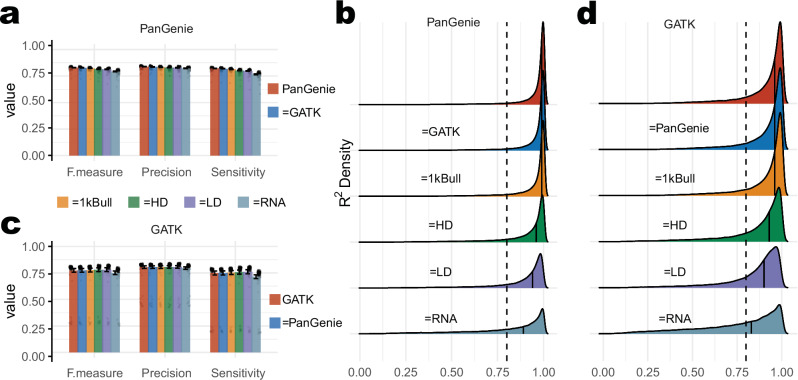
Performance of the Holstein pangenome variation imputation reference panel for SV imputation. **a** Average F1 score, precision, and sensitivity of SV imputation using PanGenie SNPs across four SNP density levels (1kBull, HD, LD, RNA) for 173 WGS samples. Bars represent the mean, and error bars indicate the 95% confidence interval assuming a normal distribution. **b** Beagle *R*² density distribution of SV loci using PanGenie SNPs, compared with GATK SNPs and downsampled SNP density levels. **c** Average F1 score, precision, and sensitivity of SV imputation using GATK SNPs across four SNP density levels (1kBull, HD, LD, RNA) for 173 WGS samples. Bars represent the mean, and error bars indicate the 95% confidence interval assuming a normal distribution. **d** Beagle *R*² density distribution of SV loci using GATK SNPs, compared with PanGenie SNPs and downsampled SNP density levels. Overall, both PanGenie and GATK SNPs achieved high SV imputation accuracy, with PanGenie SNPs showing slightly higher F1 scores, precision, sensitivity, and tighter *R*² distributions across SNP density levels. Bars represent the mean, and error bars indicate the 95% confidence interval calculated assuming a normal distribution in the a and c panels. A total of 93,059 SVs for 1302 Holstein-related cattle are included in this analysis.

PanGenie SNPs produced consistently high F1, precision, and sensitivity across SNP densities, with F1 values exceeding 0.80 for all but RNA SNPs (F1 = 0.79) (Fig. 3a and Supplementary Data 26). The DR² distributions were strongly skewed toward 1.0 for high-density inputs (1kBull, HD, PanGenie full set), indicating high-quality dosage imputation at the locus level (Fig. 3b and Supplementary Data 27). Lower-density inputs (LD, RNA) showed broader DR² distributions, but still achieved reliable accuracy, with most SVs reaching DR² ≥ 0.8.

To quantify imputation confidence, we measured the proportion of SVs with DR² ≥ 0.8. The rates increased with SNP density: RNA (64.2%), LD (81.0%), HD (83.5%), and 1kBull (91.4%), with the full PanGenie SNP set (11,934,283 SNPs) achieving 93.6%. When using PanGenie SNPs downsampled to the same density levels as GATK SNPs (11,492,334 SNPs), the rate was 92.9% (Supplementary Data 28).

### Testing with SNPs from GATK

We also applied the mapping-based GATK method for SNP calling to compare with the k-mer-based strategy. Using the 11,492,334 overlapping SNPs between PanGenie and GATK, we evaluated SV imputation performance under the same settings. GATK SNPs were further downsampled into four density categories: 1kBull (12,842,651 SNPs), HD (606,440 SNPs), LD (68,557 SNPs), and RNA (1,628,389 SNPs) densities.

SV Imputation using GATK SNPs showed strong performance, with F1 scores exceeding 0.75 across all densities and only a slight reduction in sensitivity at the RNA level (Fig. 3c and Supplementary Data 26). DR² distributions were generally consistent across densities, though marginally broader than those obtained using PanGenie SNPs, particularly at lower densities (Fig. 3d and Supplementary Data 27). The proportion of SVs with DR² ≥ 0.8 followed a similar trend to PanGenie: RNA (53.4%), LD (74.3%), HD (76.8%), 1kBull (85.2%), with the full GATK SNP set (16,135,920 SNPs) at 85.4%. When GATK SNPs were downsampled to match the PanGenie SNP density levels (11,492,334 SNPs), the corresponding DR² ≥ 0.8 rate was 85.7% (Supplementary Data 28).

These results confirm the robustness of the HolPIP for SV imputation using both k-mer-based and mapping-based SNP sources and demonstrate the panel’s applicability across a range of SNP densities in large-scale cattle genomics.

### Imputed SV-based GWAS for 38 traits in 50,299 Holstein bulls

Using HolPIP as a reference panel, we successfully imputed 68,354 high-quality SVs, including 15,950 DELs, 27,717 INSs, and 24,687 COMPLEXs from 8,130,306 SNPs in 50,299 Holstein bulls. The retention rates of SVs are 86.65% (from 78,886 SVs with MAF greater than 0.01), 88.69% (17,984, DEL), 87.23% (31,774, INS), and 84.75% (29,128, COMPLEX) under a strict threshold of Beagle DR² larger than 0.8 (Supplementary Data 29). Based on the 68,354 high-confidence SVs, we performed GWAS for 38 traits, spanning five major trait categories: Conformation and Type (CT, *n* = 19), Fertility and Calving (FC, *n* = 9), Longevity and Health (LH, *n* = 4), and Production and Yield (PY, *n* = 5), Net Merit (NM, *n* = 1) using SLEMM with a linear mixed model^40^ (Supplementary Data 30). We detected 1225 significant SV-trait associations at a genome-wide significance threshold of 7.31 × 10^−7^ for these 38 traits (Supplementary Data 31), involving 509 unique SVs (115 DEL, 191 INS, and 203 COMPLEX events). Among these, 231 SVs were mapped to 137 genes, and 126 SVs overlapped with enhancers or promoters active in eight cattle tissues (Supplementary Data 32 and Supplementary Data 33).

Notably, more than 100 SVs were detected to be significantly associated with Protein Percentage (PP, 126 SVs) and Sire Calving Ease (SCE, 115 SVs) (Supplementary Data 34). Among these SVs, 24 were significantly associated with at least 10 traits, though most of them were either unmapped or mapped to *LOC* genes of unknown functions (e.g., *LOC112442366*, *LOC100847180*, *LOC100138951*), except for a 79-bp INS on chr14 (14:874,981:875,060-INS) located within the *PLEC* gene and annotated as an enhancer in adipose tissue (Supplementary Data 35). Grouping traits revealed several multi-trait associated SVs: six SVs in a 2 Mb region at the start of chr14, near five genes (*PLEC, EPPK1, LOC112441461, TSNARE1*, and *LOC789384*), were significantly associated with all five PY traits; another six SVs in the same region near *ZNF16*, *LOC112441461*, *LOC100848939*, and *TSNARE1* were associated with all four LH traits; an 8581-bp COMPLEX SV on chr18 (~60 Mb) was significantly associated with all four LH traits, although no gene or regulatory annotation was found in the region; and the previously mentioned 79-bp INS in *PLEC* was also significantly associated with three LH traits: Livability (LIV), Somatic Cell Score (SCS), and Gestation Length (GL).

We highlight representative traits with strong signals from SV-based GWAS, including stature, milk yield, fat yield, protein percentage, and body depth. For example, a 6179 bp deletion within the *MATN3* gene was strongly associated with stature and may disrupt both coding and regulatory sequences involved in bone development (Supplementary Fig. 7a-c, Supplementary Data 29, Supplementary Data 31, Supplementary Data 32, Supplementary Data 33, and Supplementary Data 36). Additionally, a 1548 bp insertion in *EPPK1* showed robust associations with milk production traits and overlapped both coding regions and tissue-specific enhancers. For milk, fat, and protein yield, we identified several SVs clustered within a ~2 Mb region on chr14 near *PLEC*, *EPPK1*, and *ZNF250*, which is a well-known major QTL region of *DGAT1*. These SVs are likely to influence gene expression or transcript structure through coding sequence disruptions or overlap with regulatory elements (Supplementary Fig. 7d–g, Supplementary Fig. 8a–f, Supplementary Data 29, Supplementary Data 31, and Supplementary Data 32). For body depth, significant SV associations were observed in a region on chr18 previously linked to cattle conformation traits, which also overlapped stature-associated loci (Supplementary Fig. 8g, Supplementary Data 29, Supplementary Data 31, Supplementary Data 32, Supplementary Data 33, and Supplementary Data 37).

### Statistical fine-mapping incorporating SNP and SV

To assess the contribution of SVs to complex trait architecture, we conducted GWAS followed by fine-mapping in candidate regions using both SNPs and SVs. A total of 11,292,243 SNPs and 68,354 imputed SVs were included in the GWAS across 38 cattle traits in a cohort of 50,299 individuals. We applied a liberal threshold of *P* < 5 × 10^−5^ to capture a broad set of associated signals for downstream analysis, which identified 2594 candidate regions (Supplementary Fig. 9a, b). Fine-mapping within these regions was then performed using the forward selection algorithm implemented in BFMAP v0.65^41^.

We identified 32 SVs that reached the significance threshold (*P* < 5 × 10^−5^) and had high posterior inclusion probabilities (PIP ≥ 0.9), providing statistical evidence for their potential causality (Table 1, Supplementary Data 38, and Supplementary Data 39). Several biologically relevant associations emerged from this analysis. A 75-bp deletion (18:14,361,801-14,361,875:DEL) showed strong evidence for causality across multiple conformation traits including dairy form (*P* = 5.62 × 10^−6^, PIP = 0.979), rump width (*P* = 8.35 × 10^−10^, PIP = 1.000), and stature (*P* = 3.25 × 10^−6^, PIP = 1.000), overlapping the *ANKRD11* gene and annotated enhancers in adipose and hypothalamus tissues. An insertion (26:45,378,950-45,379,181:INS) showed associations with both fat yield (*P* = 3.74 × 10^−7^, PIP = 0.995) and protein yield (*P* = 9.83 × 10^−6^, PIP = 0.938), mapping to the *DHX32* gene involved in RNA metabolism. A 1.3-kb COMPLEX variant (19:26,953,736-26,955,033) associated with fat yield (*P* = 6.16 × 10^−6^, PIP = 0.999) was located within the *GABARAP* gene, which encodes a protein involved in autophagy and membrane trafficking. Additional candidates included a highly significant insertion (29:49,886,062-49,886,146:INS) associated with teat length (*P* = 2.25 × 10^−16^, PIP = 0.998), and a large 32.8-kb COMPLEX variant (23:26,372,599-26,405,398) associated with gestation length (*P* = 1.93 × 10^−8^, PIP = 0.995) that overlapped lung-specific promoter regions. The functional diversity of these candidates demonstrates the potential for SVs to affect traits through various mechanisms, including direct gene disruption, regulatory element modification, and pathway-specific effects.

**Table 1:** Fine-mapping SVs

ID	Traits	Category	*P*-value	PIP*	ADE	AA(0/0)	AA_SE	BB(1/1)	BB_SE	Gene	Enhancer/Promoter
1:22723502:22734850-COMPLEX	Fat Yield	PY	1.22E-07	1.00	8.78	−6.22	1.53	−0.12	10.01		
1:712602:713304-COMPLEX	Foot Angle	CT	4.62E-09	1.00	−0.40	0.35	0.08	−0.44	0.15		
1:712602:713304-COMPLEX	Front Teat Placement	CT	2.12E-06	1.00	−0.28	0.35	0.07	−0.20	0.14		
1:712602:713304-COMPLEX	Udder Cleft	CT	4.57E-06	1.00	−0.31	0.39	0.06	−0.72	0.10		
1:76332990:76335608-COMPLEX	Heifer Conception Rate	NM	3.86E-05	1.00	−1.13	1.01	0.27	−10.10	6.38		
10:25225248:25225968-COMPLEX	Final Score	CT	2.39E-07	1.00	−0.10	0.09	0.02	−0.19	0.10		
14:138044:138188-COMPLEX	Somatic Cell Score	NM	1.10E-05	1.00	0.01	−0.01	0.00	0.00	0.00		
14:266639:266723-COMPLEX	Fat Percentage	PY	6.83E-65	1.00	−0.03	0.04	0.00	−0.04	0.00	*ZNF16*	
14:266639:266723-COMPLEX	Protein Percentage	PY	8.72E-42	1.00	−0.01	0.01	0.00	−0.01	0.00	*ZNF16*	
14:28804830:28805099-DEL	Udder Cleft	CT	5.70E-08	0.95	0.12	−0.12	0.03	0.10	0.02		
14:66461630:66461686-DEL	Rear Udder Width	CT	5.98E-08	1.00	0.09	−0.10	0.07	0.08	0.02		
14:66461630:66461686-DEL	Final Score	CT	2.30E-07	1.00	0.17	−0.19	0.14	0.17	0.03		
14:7428546:7428757-COMPLEX	Body Depth	CT	5.67E-07	0.91	0.09	−0.09	0.02	0.09	0.02		
14:947362:948910-INS	Net Merit	NM	4.47E-05	0.91	27.99	−76.12	6.09	58.79	6.03	*EPPK1*	Adi:P,Cor:P
14:949204:950751-DEL	Protein Percentage	PY	4.87E-09	1.00	0.00	0.01	0.00	−0.01	0.00	*EPPK1*	
15:59836411:59836864-DEL	Early Calving	NM	5.51E-06	0.98	−0.83	0.57	0.24	−1.03	0.19		
18:14361801:14361875-DEL	Rump Width	CT	8.35E-10	1.00	−0.13	0.17	0.03	−0.10	0.04	*ANKRD11*	Adi:E,Cor:P,Hyp:E,Mus:P
18:14361801:14361875-DEL	Stature	CT	3.25E-06	1.00	−0.17	0.14	0.04	−0.20	0.05	*ANKRD11*	Adi:E,Cor:P,Hyp:E,Mus:P
18:14361801:14361875-DEL	Dairy Form	CT	5.62E-06	0.98	−0.20	0.22	0.04	−0.16	0.05	*ANKRD11*	Adi:E,Cor:P,Hyp:E,Mus:P
18:27687882:27696433-COMPLEX	Productive Life	NM	1.38E-09	1.00	0.78	−0.74	0.12	0.73	0.42		
18:61240250:61242138-DEL	Daughter Calving Ease	LH	4.01E-07	0.94	0.07	−0.04	0.01	0.04	0.02	*MGC138914*	
18:61245855:61247049-COMPLEX	Gestation Length	LH	1.61E-07	0.98	0.08	−0.06	0.02	0.08	0.02	*MGC138914*	
18:62476934:62477005-COMPLEX	Sire Calving Ease	LH	3.21E-07	0.92	0.06	−0.04	0.01	0.05	0.01	*LOC112442414*	
18:7364638:7364924-COMPLEX	Net Merit	NM	8.56E-06	0.99	39.24	−12.37	8.50	16.32	11.19		
19:26953736:26955033-COMPLEX	Fat Yield	PY	6.16E-06	1.00	−3.94	3.81	0.86	−1.16	1.75	*GABARAP*	
2:134049818:134049912-INS	Sire Stillbirth Rate	LH	1.86E-06	0.96	−0.08	0.05	0.02	−0.10	0.02		
2:16515486:16515555-INS	Dairy Form	CT	5.85E-06	1.00	0.14	−0.10	0.03	0.01	0.07		
23:26372599:26405398-COMPLEX	Gestation Length	LH	1.93E-08	1.00	−0.21	0.16	0.04	−0.21	0.04		Lun:P
23:48191273:48191431-DEL	Sire Stillbirth Rate	LH	3.17E-06	1.00	0.14	−0.12	0.03	0.39	0.17	*RREB1*	Adi:E
25:40695695:40695780-INS	Front Teat Placement	CT	3.78E-07	0.90	−0.15	0.12	0.03	−0.15	0.05	*IQCE*	
26:45378950:45379181-INS	Fat Yield	PY	3.74E-07	0.99	3.41	−3.37	0.70	3.20	0.72	*DHX32*	Mus:E
26:45378950:45379181-INS	Protein Yield	PY	9.83E-06	0.94	2.23	−2.57	0.52	1.28	0.53	*DHX32*	Mus:E
29:49886062:49886146-INS	Teat Length	CT	2.25E-16	1.00	−0.14	0.05	0.02	−0.09	0.02	*LOC112444885*	
3:7273500:7274582-DEL	Cow Conception Rate	NM	1.93E-08	1.00	−0.62	0.30	0.10	−0.26	0.18	*NOS1AP*	
5:118395967:118396063-COMPLEX	Final Score	CT	1.88E-05	0.98	−0.05	0.04	0.01	−0.05	0.02	*TAFA5*	Adi:P,Liv:P
5:118825822:118825890-INS	Rear Udder Height	CT	5.64E-06	1.00	0.09	−0.02	0.03	0.04	0.02		
7:13454881:13471359-COMPLEX	Fore Udder Attachment	CT	6.39E-06	0.92	0.14	−0.13	0.03	0.10	0.04	*OR7G8,OR7G50*	Adi:P
9:1217099:1225651-COMPLEX	Productive Life	NM	2.75E-05	1.00	0.66	−0.19	0.13	0.54	0.71		
9:78899490:78900559-INS	Protein Yield	PY	2.22E-05	0.99	−2.78	1.72	0.63	−1.64	0.94		

*PIP: Posterior inclusion probability of the variant being causal. ADE: Additive genetic effect estimate from the GWAS model. **AA (0/0)**: Estimated effect size for the homozygous reference genotype. **AA_SE:** Standard error of the effect estimate for the homozygous reference genotype. **BB (1/1):** Estimated effect size for the homozygous alternative genotype. **BB_SE:** Standard error of the effect estimate for the homozygous alternative genotype.

*CT* conformation and type, *FC* fertility and calving, *LH* longevity and health, *PY* production and yield, *NM* net merit.

*P* promoter, *E* enhancer, *Adi* adipose, *Cor* cortex, *Hyp* hypothalamus, *Mus* muscle, *Lun* lung.

### Functional enrichment analysis for genome-wide polygenic effects of SV

#### Small-effect enrichment analysis

To evaluate the contribution of genome-wide SVs beyond large-effect QTL signals, we applied MPH with fine-mapped lead variants as fixed effects to the partition method signals. Comparing models with and without SVs, the SV component was statistically significant in 20 of 38 traits (likelihood ratio test, FDR-adjusted *P* < 0.05), indicating that incorporating SVs improved model fit for these traits. However, we observed an average increase of only 0.11% in genetic variance explained across 38 traits beyond that captured by the SNP-only model (Supplementary Data 40), suggesting limited practical improvement in genomic prediction accuracy from incorporating SVs into models.

**Fig. 4:**
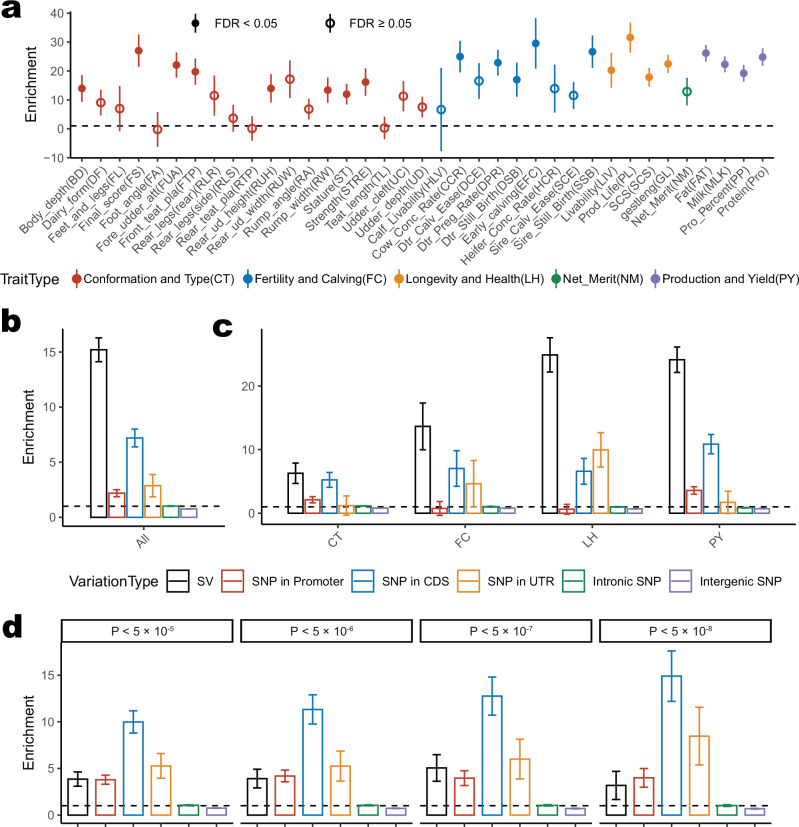
Functional enrichment analysis of fine-mapped variants by trait category, variant type, and significance threshold. **a** Enrichment of SVs across individual traits for a total of 50,299 cattle, grouped by 5 trait categories: Conformation and Type (CT), Fertility and Calving (FC), Longevity and Health (LH), Net Merit (NM), and Production and Yield (PY). Circles indicate mean enrichment estimates. MPH enrichment represents the relative contribution of a given variant category to mid-parent heterosis compared with the genome-wide expectation. An enrichment value greater than 1 indicates that this category contributes disproportionately to MPH. Error bars denote the standard error (SE) of the enrichment estimate, reflecting uncertainty due to sampling and genomic variation. **b** Average enrichment across all 13 selected traits for 6 annotation categories from left to right: SVs, SNPs in promoter regions, CDS, UTRs, intronic, and intergenic regions. SE error bars of the enrichment were estimated by resampling genomic segments using a block jackknife approach, which accounts for linkage disequilibrium and uneven variant distribution across the genome. **c** Average enrichment per trait group (CT, FC, LH, PY) across 13 selected traits for 6 annotation categories. SE error bars of the enrichment were estimated by resampling genomic segments using a block jackknife approach. **d** Enrichment of variant categories under progressively stringent GWAS significance thresholds (*P* < 5 × 10^−5^ to *P* < 5 × 10^−8^), highlighting trends across 13 selected traits for 6 annotation categories. Genomic enrichment represents the relative contribution of a given variant category to the overall genetic effect compared with the genome-wide expectation. An enrichment value greater than 1 indicates that this category is overrepresented in explaining genetic effects. Error bars denote the SE of the enrichment estimate obtained using a block jackknife resampling approach. In all panels, bars and points represent model-derived enrichment estimates rather than sample means, and the error bars represent uncertainty in those estimates. Consequently, no underlying observation-level data distributions exist for overlay. *P*-value is not derived from a hypothesis test but is used solely as a classification threshold in the GWAS analysis to define statistical significance.

We investigated the enrichment of genetic variants across functional annotation categories using the 13 selected largely independent traits (see “Materials and Methods”). SNPs were categorized by functional annotation (e.g., CDS, UTRs, promoters, intronic, intergenic), while SVs were treated as a single category. Our results revealed that SVs consistently exhibited greater enrichment than SNPs when analyzing the polygenic contributions to trait variation (Fig. 4a and Supplementary Data 41). Specifically, SV enrichment was particularly pronounced for traits such as Productive Life (31.57×), Early Calving (29.54×), Cow Conception Rate (24.98×), Fat Yield (26.16×), Protein Yield (24.81×), and Milk Yield (22.30×). Notable enrichments were also observed for fertility, health, and conformation traits, including Daughter Stillbirth (16.99×), Daughter Calving Ease (16.55×), Strength (16.17×), Rump Width (13.38×), and Stature (11.98×). Collectively, these enrichments significantly exceeded those observed for genome-wide SNPs, highlighting the substantial and distinctive contribution of SVs to the polygenic architecture of economically important cattle traits.

Across all analyzed traits (Fig. 4b, Supplementary Fig. 9c, and Supplementary Data 42), SVs showed an average enrichment of 15.20×, significantly surpassing enrichment observed in other annotation groups such as CDS (7.20×), UTRs (2.87×), promoters (2.19×), intronic (1.02×), and intergenic SNPs (0.76×). Trait-specific analysis highlighted striking SV enrichments, particularly in categories of Longevity and Health (24.89×) and Production and Yield (24.13×), further emphasizing the disproportionate functional contribution of small-effect SVs compared to traditional SNP variants. Enrichment in the Fertility and Calving group was also substantial (13.65×), underscoring the pervasive influence of SVs on polygenic architecture across diverse cattle traits (Fig. 4c).

These results demonstrate that SVs represent a functionally important component of cattle genomic variation, exhibiting substantially higher enrichment than SNPs across functional annotations among small-effect variants. The pronounced enrichment across economically important trait categories indicates that SVs may be particularly valuable for understanding biological mechanisms underlying complex traits.

#### Large-effect enrichment analysis

We applied GEMRICH analysis (https://github.com/jiang18/gemrich) to further explore the enrichment of fine-mapped large effects across different genomic annotation categories at varying *P*-value thresholds (*P* < 5 × 10^−5^ to *P* < 5 × 10^−8^). Due to the limited number of fine-mapped SVs per individual trait, we performed a meta-analysis for the four trait groups (PY, CT, LH, and FC from 13 unrelated traits).

Overall, SNPs located in CDS consistently exhibited the highest enrichment, increasing progressively from 9.99× at *P* < 5 × 10^−5^ to 14.90× at the most stringent threshold (*P* < 5 × 10^−8^) (Fig. 4c and Supplementary Data 43). SNPs in untranslated regions (UTRs) showed intermediate enrichment, reaching up to 8.47× at *P* < 5 × 10^−8^, followed by SNPs in promoter regions (3.99× at *P* < 5 × 10^−8^). SVs also demonstrated notable enrichment, intermediate between promoter SNPs and UTR SNPs, ranging from 3.86× at *P* < 5 × 10^−5^ to 3.18× at *P* < 5 × 10^−8^. In contrast, SNPs in intronic and intergenic regions displayed minimal or no enrichment across all significance thresholds.

Trait-specific analyses further clarified these trends, with substantial enrichment for CDS SNPs observed at the most stringent threshold (*P* < 5 × 10^−8^) for traits from Production and Yield (24.19×), Longevity and Health (20.57×), Fertility and Calving (9.31×), and Conformation and Type (8.33×) (Supplementary Data 44). SV enrichment also varied significantly by trait category, with particularly high enrichment in Longevity and Health (10.54× at *P* < 5 × 10^−8^) and Fertility and Calving (5.73× at *P* < 5 × 10^−7^), underscoring their distinct and substantial contributions compared to most SNP categories. These results reinforce the functional significance of variants identified by GEMRICH, highlighting the critical roles of both coding SNPs and SVs in shaping complex cattle traits.

SV subtypes also exhibited substantial enrichment, ranking between SNPs in CDS and promoter regions (Supplementary Fig. 9d and Supplementary Data 44). COMPLEX events showed the highest enrichment, peaking at 6.67× (*P* < 5 × 10^−7^), followed closely by DELs (5.65× at *P* < 5 × 10^−6^) and INSs (4.96× at *P* < 5 × 10^−5^). These findings underscore the pronounced functional impact of COMPLEX, DEL, and INS variants, positioning SV subtypes as crucial contributors that bridge coding-region SNPs and other regulatory annotations.

## Discussion

We implemented and evaluated genome-wide SV imputation using pangenome graphs in livestock, achieving high imputation accuracy across the genome. Furthermore, with a cohort size more than ten times larger than that of comparable human studies (e.g., Sirén et al.^13^), it represents one of the largest SV-based GWAS analyses reported to date in any animal, second only to another large-scale UK Biobank human study currently under review^15^. However, in that human study, data coverage was admittedly limited for the use of pangenomes for SV imputation^15^. The key contribution of this study lies in extending the use of pangenomes for SV imputation to cattle. While both studies demonstrate comparable accuracy for population-scale SV imputation and association testing, they differ in biological system, reference framework, and analytical objectives. The human study focused on disease fine-mapping using a large linear-reference-based SV panel, whereas our work applies a pangenome graph framework to livestock and evaluates the contribution of SVs to complex production traits and genomic selection. A notable finding of our study is the superior imputation accuracy for homozygous SV genotypes (F1 = 0.93) compared to heterozygous ones (F1 = 0.84). This result likely stems from a combination of biological factors, such as selection-driven homozygous haplotypes, and technical factors, specifically the increased reliability of imputing common major alleles across the frequency spectrum. In addition, incorporating SVs also provides a more complete view of the genetic architecture of economically important traits, as SVs show strong functional enrichment despite explaining a modest fraction of additive variance. Together, these findings highlight the limitations of linear reference genomes and the practical advantages of pangenome graphs for resolving complex structural variation in cattle breeding and genetic improvement.

Our work builds on insights from existing human, sheep, plant, and other pangenome studies^15–28^ by extending pangenome-based SV genotyping into a large-scale livestock setting that had previously lacked comparable resources. Our study highlights the power of pangenomic approaches in uncovering the genetic diversity that drives key traits in livestock, with a focus on Holstein cattle. By constructing the Holstein pangenome variation imputation reference panel (HolPIP), we conducted a genome-wide SV imputation in livestock, imputing over 68,000 SVs with high accuracy. Leveraging this resource, we performed large-scale SV-based GWAS in more than 50,000 animals, identifying SV-trait associations related to economically important traits across four key categories: Conformation and Type, Production and Yield, Longevity and Health, and Fertility and Calving.

While earlier studies demonstrated the feasibility of pangenome-based SV detection and imputation, our contribution lies in translating these concepts into a cattle system at an unprecedented scale and in providing a publicly usable imputation resource for the dairy genomics community. Importantly, we deliver a practical, data-driven evaluation of how imputed SVs influence genomic analyses by testing whether they improve trait-association signals and increase the proportion of explained genetic variance. Although the gain was modest, this finding is itself informative, clarifying current limitations such as imputation accuracy, allele frequency spectra, and SNP tagging, and guiding future improvements in sequencing depth, breed representation, and imputation strategy. These results provide a comprehensive view of SV diversity in the cattle genome and establish a foundation for future incorporation of SVs into genomic selection and genetic improvement programs.

Our findings underscore the advantages of using breed-specific pangenome references for SV genotyping and imputation in Holsteins. We note that while multi-breed pangenomes would be ideal for cross-breed applications, our Holstein-specific panel performed surprisingly well on Jersey samples. This exploratory test suggests that shared common SVs can be accurately captured across breeds, though we emphasize that these results depend on variant density and allele frequency, and should be viewed as unvalidated for general cross-breed use. HolPIP nonetheless outperformed existing livestock SV imputation panels, producing higher Beagle *R*² values and a substantially greater number of imputed SVs. Crucially, our k-mer-based genotyping approach via PanGenie effectively addresses a common challenge in SV imputation: the presence of SNPs within deleted sequences. While such SNPs typically lead to inaccurate results due to missing genotypes in homozygous deletion carriers and reduced allelic information in heterozygotes, our pipeline inherently excludes these variants from the reference panel. This ensures that the resulting imputation is not confounded by ‘phantom’ SNPs in deleted regions, thereby avoiding potential biases in downstream analyses. These advances extend the pangenome SV paradigm from human and small-scale livestock research into a large, multi-cohort cattle framework, offering both a valuable community resource and actionable insights for future SV-imputation studies.

We identified 1225 significant SV-trait associations across the Holstein population, highlighting the important role of SVs in phenotypic variation. Notably, a 6179 bp deletion on chr11 overlapping the *MATN3* gene, known to influence bone and cartilage development, was associated with stature^42^. This variant could serve as a valuable marker in selection programs, given the stature’s impact on body mass and feed efficiency^43,44^. Additionally, a 1548 bp insertion on chr14 overlapping *EPPK1* was strongly associated with milk fat percentage, suggesting that SVs in both regulatory and coding regions play key roles in dairy production efficiency. Given the large amount of selection now taking place for greater feed saved/lower residual feed intake (RFI) and lower body weight composite, an important question is whether these variants need to be explicitly targeted in selection, or whether ongoing selection will naturally drive them toward fixation.

Our findings demonstrate how pangenome-based analyses refine SV detection and improve imputation accuracy for genomic selection in livestock breeding. HolPIP enables high-resolution tracking of genetic variation, enhancing both the precision and sensitivity of trait-associated SV discovery. Integrating SVs into genomic prediction models offers the potential to improve genetic evaluations by capturing more functionally relevant variants, especially for complex traits. This approach has the potential to inform breeding decisions that could help manage inbreeding and preserve genetic diversity in elite dairy populations. For example, pangenome data can be used to identify and select underrepresented haplotypes or reduce reliance on highly similar sires. Furthermore, combining high-confidence SVs with SNP markers may increase heritability estimates by accounting for additional functional variation, ultimately supporting more efficient and productive cattle breeding programs.

While our study advances breed-specific SV detection using a pangenomic framework, it is limited by sample size and the exclusion of short indels (<50 bp). Despite enlarged genomic resources, some QTL, such as the region near 51 Mb on chr18^45^, remain unresolved, reflecting both biological complexity and annotation gaps. Expanding the panel with additional haplotypes, particularly from diverse populations or long-read assemblies, would likely improve the ability to impute a broader spectrum of SVs. Larger, more diverse datasets, functional validation, and integration with FarmGTEx and Ruminant T2T resources will be key to linking SVs to complex traits. Expanding pangenomic analyses to other farm-animal species will further uncover the role of SVs in animal performance and adaptation, with applications beyond dairy cattle, such as beef breeds and other livestock.

Despite some limitations, our study highlights the power of pangenomic approaches in capturing the diverse SV landscape. We demonstrate how SVs can improve genome imputation and identify functional variants associated with complex traits. Using imputed SV in a large cattle population, we report higher heritability enrichment of SV regions compared to other functional genome regions across many traits. Integrating SVs into breeding programs could enhance productivity and resilience in dairy cattle. As sequencing technologies and computational methods continue to improve, pangenomics is poised to play a pivotal role in population genetic studies, supporting precision breeding and sustainable agriculture.

## Materials and methods

### Ethics

Ethical approval for animal experimentation is not applicable to this study, as no animal experiments, interventions, or sample collection were performed. All data analyzed in this work were obtained from previously published studies and/or existing datasets with original ethical approvals granted by the respective institutions.

### Previous pangenome construction

We retrieved HiFi sequencing data for 20 Holsteins from a previous study (PRJNA1113979)^31^. The data had an average read length of 13–23 kb and coverage of 23.2–33.5× relative to the ARS-UCD v2.0 reference genome. Briefly, for de novo assembly, we utilized the Hifiasm (v.0.18.5) assembler^46^, resulting in the generation of 20 primary haploid assemblies and 40 phased diploid assemblies. To improve contig phasing accuracy, Hi-C data were generated for two selected Holstein individuals and assembled in Hifiasm’s Hi-C integration mode. For pangenome graph construction, we used the Minigraph-Cactus (MC) pipeline (v2.4.2) with the ARS-UCD v2.0 genome as the backbone^47^. MC parameters included “cactus-pangenome --binariesMode local --reference ARS_UCD_v2.0 --giraffe clip filter --gbz clip filter full --gfa clip filter full --vcf --permissiveContigFilter --haplo --chrom-vg clip filter --chrom-og full --viz”. The “--permissiveContigFilter” parameter was used to enhance sensitivity in regions with fragmented or highly diverse contigs.

### Variation genotyping for WGS data using pangenome references

Using the Holstein pangenome VCF generated with Minigraph-Cactus, we genotyped SVs from WGS data by PanGenie (v3.0.1), a short-read genotyper designed for detecting various genetic variants within a pangenome graph. PanGenie calculates genotypes by using read k-mer counts and a reference panel of known, fully assembled haplotypes. To refine the VCF, we filtered out the variations in the multi-allele graph VCF file with the following thresholds: sites within nested regions, variants over 100 kb, sites with ≥20% missing data, and those on sex or unplaced chromosomes. We decomposed bubbles to identify all nested variant alleles using a script (https://github.com/eblerjana/genotyping-pipelines/tree/main/prepare-vcf-MC). We then designated variations with sizes larger than 50 bp as PanGenie SV, while others were classified as SNV (SNP, MNP, and INDEL).

### SV catalog

To construct a pangenome-based SV panel for large-scale population analyses, we downloaded 4,682 public WGS datasets from the NCBI SRA database. Of them, we kept 1672 data from Holstein, Holstein-related, and Jersey cattle with at least 5× coverage to the ARS-UCD2.0 reference genome. After removing samples with outlier SV counts, a total of 1571 samples were retained, including 934 Holstein, 269 Jersey, and 368 Holstein-Jersey crossbreed samples. As described earlier, we genotyped variations using VCF files based on the BovHol pangenome reference by PanGenie, merged using BCFtools merge, and filtered to exclude variants with a missing rate of over 10%. PanGenie SVs (pSVs) greater than 50 bp were retained for downstream analyses.

### SV annotation

We annotated repeat- and gene-overlapping SVs using the *findOverlaps* function in the GenomicRanges R package (v1.40.0), with a minimum overlap threshold of 1 bp. As previously described^48–50^, the enrichment of specific genomic regions within SVs was assessed using fold tests with the whole genome as the background and Chi-squared tests for statistical significance. *P*-values were adjusted with the Bonferroni correction. Gene ontology (GO) and Kyoto Encyclopedia of Genes and Genomes (KEGG) pathway enrichment analyses were performed on gene lists of interest using the *enrichGO* and *enrichKEGG* functions from the clusterProfiler R package (v3.16.1, R v4.0.2), with a Bonferroni-adjusted significance threshold of 0.01.

### Population analysis

We conducted population genetic analysis on 1571 Holstein, Holstein-related, and Holstein-Jersey crossbreed cattle using three datasets: mapping-based DeepVariant SNVs (dSNVs)^51^, pangenome-based PanGenie variations (pVar), and PanGenie SV (pSV). After removing markers with MAF < 0.01, we performed LD pruning with PLINK (v1.9) to remove variants using parameters --indep-pairwise 50 5 0.2^52^. To investigate population structure, we performed principal component analysis (PCA) for each dataset (dSNVs, pVar, and pSV) using PLINK (v2.0). Pairwise fixation index (*F*_*ST*_) values between Holstein and Jersey breeds for common markers (MAF ≥ 0.01) were computed using VCFtools (v0.1.16). High *F*_*ST*_ markers were identified to have the top 1% value.

### Imputation analysis

We performed the imputation tests on each of the variation types detected by PanGenie for 1571 cattle, including SNP, MNP, INDEL, SNV (SNP, MNP, and INDEL), INS, DEL, and COMPLEX. Accuracy was estimated by 10× cross-validation, where each test set comprised 10% of samples with the remaining 90% as training data. We randomly introduced missing values at different rates (0%, 20%, and 50%) for each sample. In addition, we set all SVs (≥50 bp) as missing values for testing the accuracy of SV imputation by short variations (SNP, MNP, or INDEL) only. Each of the allele genotypes was evaluated separately, including REF (0/0), ALT (0/1 or 1/1), HET (0/1), HOM (1/1), and ALL. We assessed the effects of MAF (thresholds: 0.01, 0.05, and 0.1), breed, and sample size by selecting subsets of 250 Holsteins, 250 hybrids, 250 Jerseys, 750 Holsteins, and 750 samples (250 Holsteins, 250 Hybrid samples, and 250 Jerseys). We compared the performance of the two tools: Minimac4 (v4.1.6) and Beagle (v5.4). Precision, sensitivity, and F-measure were calculated by RTG Tools (v3.12.1) vcfeval function for both original genotypes and imputed genotypes. We retained one SNP per 1000 base pairs using VCFtools (v0.1.16) with the command --thin 1000.

### SNP density-based downsampling

To evaluate the impact of SNP density on SV imputation performance, we constructed multiple SNP panels representing a gradient of marker densities. SNPs were extracted based on their genomic positions from the WGS dataset of the 1000 Bulls Project (Run 8). The following panels were defined: (1) 1kBullSNP: a high-density panel with 15,758,693 SNPs genome-wide^53^; (2) hdSNP: corresponding to the BovineHD BeadChip array (777 K), comprising 774,316 SNPs; (3) ldSNP: representing the BovineSNP50 array, with 68,706 SNPs^54^; (4) RNA-SNP: consisting of 1,451,919 SNPs derived from RNA-seq data.

The RNA-SNP set was generated from 19 Holstein blood samples matched with PacBio HiFi sequencing data (one sample was excluded due to missing data), and intersected with SNPs identified in the FarmGTEx-Cattle dataset^39^.

All SNP subsets were extracted using their chromosomal coordinates to match the SV genotyping framework. These density-based SNP sets were then integrated into SV imputation models using the Beagle phasing and imputation software, with performance metrics evaluated across varying marker densities.

### Phenotypes data

Individual trait data for ~90% of 173 registered and 50,299 Holstein bulls were retrieved from the December 2021 genomic evaluations of the U.S. Council of Dairy Cattle Breeding (CDCB). These 38 phenotypes included predicted transmitting ability (PTA) values for: body depth (BD); cow conception rate (CCR); cow livability (LIV); dairy form (DF); daughter pregnancy rate (DPR); daughter calving ease (DCE); daughter stillbirth (DSB); early first calving (EFC); fat percentage (FP), fat yield (FAT); feet and leg composite (FLC); final score (FS); foot angle (FA); fore udder attachment (FUA); front teat placement (FTP); gestation length (GL); heifer conception rate (HCR); heifer livability (HLV); milk yield (MLK); net merit (NM), productive life (PL); protein percentage (PP), protein yield (PRO); rear legs-rear view (RLR); rear legs-side view (RLS); rear teat placement (RTP); rear udder height (RUH); rear udder width (RUW); rump angle (RA); rump width (RW); sire calving ease (SCE); sire stillbirth (SSB); somatic cell score (SCS); stature (ST); strength (STR); teat length (TL); udder cleft (UC); and udder depth (UD). De-regressed PTA (dPTA) values for each trait were calculated as described before^55^, to remove the contribution of parent information and reduce dependence among animals. This process generated a dPTA value and its corresponding reliability for each bull, reflecting the amount of information from individual performance and progeny records. Since dPTAs are derived from phenotypes of large numbers of daughters for each bull, they exhibit substantially higher reliability than individual cow phenotypes, effectively increasing the pseudo-heritability and thereby enhancing association detection compared to analyses using individual animal records, which is directly proportional to the product of sample size and trait heritability^56^. For example, for milk fat, the minimum and mean numbers of daughters per bull are 37 and 2764 in the 173-bull dataset, and 10 and 1106 in the 52,099-bull dataset.

### Genome-wide association study between SV and phenotypes

With the Holstein pangenome variation imputation reference panel (HolPIP), we imputed SVs by SNP using Beagle (v5.4). After filtering with MAF larger than 0.01 and Beagle *R*² larger than 0.8, we conducted GWAS on the high-confidence SVs. The analysis covered 38 cattle traits using SLEMM (Stochastic-Lanczos-Expedited Mixed Models) with a linear mixed model with a significance threshold of 7.31 × 10^−7^ for 68,354 common SVs, serving as an initial discovery step for identifying SV-trait associations.

### Pangenome SV analyses for 173 whole-genome sequencing samples

Employing PanGenie (v3.0.1), we genotyped the pangenome SVs from 173 whole-genome sequenced (WGS) samples based on the Holstein pangenome reference H20D. These samples served as an independent validation set for evaluating the imputation performance of HolPIP.

### Fine-mapping and variant selection

To assess the contribution of SVs to complex trait architecture, we conducted joint genome-wide association and fine-mapping analyses across 38 cattle traits in a cohort of 50,299 individuals. The analysis incorporated 11,292,243 SNPs and 68,354 imputed SVs. A liberal significance threshold of *P* < 5 × 10^−5^ was applied to capture associated signals from the GWAS. Candidate regions for fine-mapping were then defined following our previous procedure^41,57^, where each region spanned at least 2 Mb and covered all significant variants. Fine-mapping within these candidate regions was performed using the forward selection algorithm implemented in BFMAP v0.65 (Jiang et al.^41^), which sequentially adds independent signals to the model, repositions them for refinement, and identifies variants related to each signal. Variants within signals whose lead variant reached genome-wide significance (*P* < 5 × 10^−8^) and that had high posterior inclusion probability (PIP ≥ 0.9) were considered high-confidence candidate variants.

### Functional enrichment of genetic effects

In this analysis, Large effects refer to significant effects detected by GWAS, while small effects represent the remaining polygenic effects.

To evaluate the contribution of small-effect variants beyond lead GWAS signals, we applied the MPH v0.52.1^58^, which partitions genetic variance across functional annotations using restricted maximum likelihood. Lead variants (SNPs or SVs) from each fine-mapped signal were included as fixed effects to control for large-effect loci, allowing estimation of the polygenic background variance attributable to small-effect variants. SNPs were partitioned by comprehensive functional annotations, while SVs were treated as a single category.

The MPH approach partitions total genetic variance into components corresponding to different functional categories using linear mixed models, as shown below:

1 $$\begin{array}{c}{{\bf{y}}}={{\bf{X}}}{{\mathbf{\beta }}}\,+\,{{\bf{Z}}}{{\boldsymbol{\alpha }}}\,+\,{{\bf{e}}}\\ {{\boldsymbol{\alpha }}}\, \sim \,{{\mathscr{N}}}\left(0,{{\bf{D}}}\right)\\ {{\bf{e}}}\, \sim \,{{\mathscr{N}}}\left(0,{{\bf{R}}}{\sigma }_{e}^{2}\right)\end{array}$$

where **y** is a vector of dPTA for a trait in a sample of *N* individuals; **β** is a vector of fixed effects used to account large effect; **X** is the design matrix for **β**; **Z** is a matrix of standardized genotypes for *N* individuals and *M* SNPs; **α** is a vector of additive genetic effects for *M* SNPs; **D** is a diagonal matrix with the *j*^th^ diagonal element being the additive genetic variance of SNP *j*; and **e** is a vector of residuals. ${\sigma }_{e}^{2}$ is the residual variance component, and **R** models differential reliabilities as ${R}_{{ii}}=1/{{r}_{i}}^{2}-1$, where ${{r}_{i}}^{2}$ is the reliability of the *i*-th individual’s dPTA.

### Small-effect enrichment

We compared models with and without SVs to assess the additional variance contribution of SVs beyond that captured by SNPs alone. Enrichment of functional annotations was calculated based on partitioned variance results from MPH. This enrichment was calculated as the ratio of the average genetic variance explained per variant within each functional annotation category to the average genetic variance explained per variant genome-wide. By definition, the heritability enrichment of whole-genome variants is exactly 1. To enable valid inverse-variance weighted meta-analysis across trait categories, we tried to avoid highly correlated traits within each group based on genetic correlation estimates, resulting in 13 independent traits. The PY group included Milk Yield and Fat Yield; the CT group included Strength, Dairy Form, Rump Angle, Udder Depth, Teat Length, Feet and Legs, and Rear Teat Placement; the LH group included Productive Life and Gestation Length; and the FC group included Sire Calving Ease and Daughter Stillbirth.

### Large-effect enrichment

To further assess enrichment patterns of large effects, we applied GEMRICH to fine-mapped variants under different *P*-value thresholds (*P* < 5 × 10^-5^ to *P* < 5 × 10^-8^). Only fine-mapped signals with lead variant *P*-values below each significance threshold were included in the analysis. GEMRICH performs maximum likelihood estimation to calculate the probability of causal variants residing within each functional annotation category, using posterior inclusion probabilities from fine-mapping summary statistics. Enrichment is estimated as the ratio of the probability of causal variants being in each annotation category to the genome-wide proportion of variants in that category. Due to the limited number of significant SVs per trait, we conducted meta-analysis by combining fine-mapping summary statistics within each of the four major trait groups: Production and Yield, Conformation and Type, Longevity and Health, and Fertility and Calving. This approach enabled assessment of how functional enrichment patterns varied across different *P*-value cutoffs relative to genome-wide expectations.

### Reporting summary

Further information on research design is available in the Nature Portfolio Reporting Summary linked to this article.

## Supplementary information

Supplementary Figs..pdf

Description of Additional Supplementary Files

Supplementary Data1

Supplementary Data2

Supplementary Data3

Supplementary Data4

Supplementary Data5

Supplementary Data6

Supplementary Data7

Supplementary Data8

Supplementary Data9

Supplementary Data10

Supplementary Data11

Supplementary Data12

Supplementary Data13

Supplementary Data14

Supplementary Data15

Supplementary Data16

Supplementary Data17

Supplementary Data18

Supplementary Data19

Supplementary Data20

Supplementary Data21

Supplementary Data22

Supplementary Data23

Supplementary Data24

Supplementary Data25

Supplementary Data26

Supplementary Data27

Supplementary Data28

Supplementary Data29

Supplementary Data30

Supplementary Data31

Supplementary Data32

Supplementary Data33

Supplementary Data34

Supplementary Data35

Supplementary Data36

Supplementary Data37

Supplementary Data38

Supplementary Data39

Supplementary Data40

Supplementary Data41

Supplementary Data42

Supplementary Data43

Supplementary Data44

Reporting Summary

Transparent Peer Review file

## Source data

SourceData.zip

## Data Availability

The data that support the results of this research are available within the article and its Supplementary Information files. Pangenome graph files (GFA) and the corresponding biallelic structural variant annotation files (anno_biallelic.vcf.gz) for the Holstein pangenome assembly (H20D) were generated and are publicly available via Zenodo under accession 10.5281/zenodo.15065028 [https://zenodo.org/records/15065028]. All sequencing and phenotypic metadata were systematically curated to ensure consistency across datasets before SV discovery, population genetic analyses, imputation, and genome-wide association studies. To construct a comprehensive pangenome-based SV catalog, we downloaded 4682 publicly available cattle whole-genome sequencing (WGS) datasets from the NCBI Sequence Read Archive. A complete list of all WGS accession numbers (including NCBI BioSample and Biopoject) is provided in Supplementary Data File 1. After applying coverage and quality control filters (≥5× coverage relative to the ARS-UCD v2.0 reference genome and removal of samples with outlier SV counts), 1571 WGS samples were retained, comprising 934 Holsteins, 269 Jerseys, and 368 Holstein–Jersey crossbred individuals. These samples were used for pangenome-based variant genotyping, population genetic analyses, and benchmarking of SV imputation performance. The Holstein pangenome variation imputation reference panel (HolPIP) is freely available under the “Genotype imputation panel” at https://cattlecellgtex.farmgtex.org/download and has also been deposited in Zenodo under accession 10.5281/zenodo.15065028 [https://zenodo.org/records/15065028]. For large-scale phenotype association analyses, trait records for 173 registered Holstein bulls with WGS data and 50,299 Holstein bulls genotyped using SNP arrays were obtained from the December 2022 genomic evaluations of the U.S. Council on Dairy Cattle Breeding (CDCB, official application link https://uscdcb.com/data-request). A total of 38 economically important production, fertility, health, and conformation traits were analyzed, with de-regressed predicted transmitting ability (dPTA) values calculated to reduce parental information bias and enhance statistical power. The December 2022 genomic evaluations produced by the Council on Dairy Cattle Breeding (CDCB) are not accessible as a single, public dataset for unrestricted download. To access restricted CDCB genomic data, you must complete the official CDCB Data Request Form (https://uscdcb.com/data-request/), which triggers a review process where research requests are evaluated by the Research Advisory Group (RAG) and Board, while commercial requests are reviewed by the CEO. Approved access typically requires signing a formal Material Transfer Agreement (MTA) or service agreement, with data often delivered exclusively through CDCB server; timelines vary based on request complexity and the necessary legal oversight. Source data are provided with this paper.

## Code availability

Additional scripts and resources are available on GitHub at https://github.com/xyxss/cattlePanSVimp, and the SV association analysis pipeline is available at https://github.com/JJWang259/sv-association-pipeline. To ensure permanent accessibility, an archived version of this repository has been deposited in Zenodo under accession 10.5281/zenodo.15065028 [https://zenodo.org/records/15065028].
