# Supplementary material for: Pangenome-based structural variant imputation enables large-scale genotype-phenotype studies in dairy cattle
Source: Nat Commun. 2026 Jul 3;17:8281. doi: 10.1038/s41467-026-75219-x (PMC13469606; doi:10.1038/s41467-026-75219-x)
Supplement: Supplementary file 1 [file 41467_2026_75219_MOESM1_ESM.pdf]

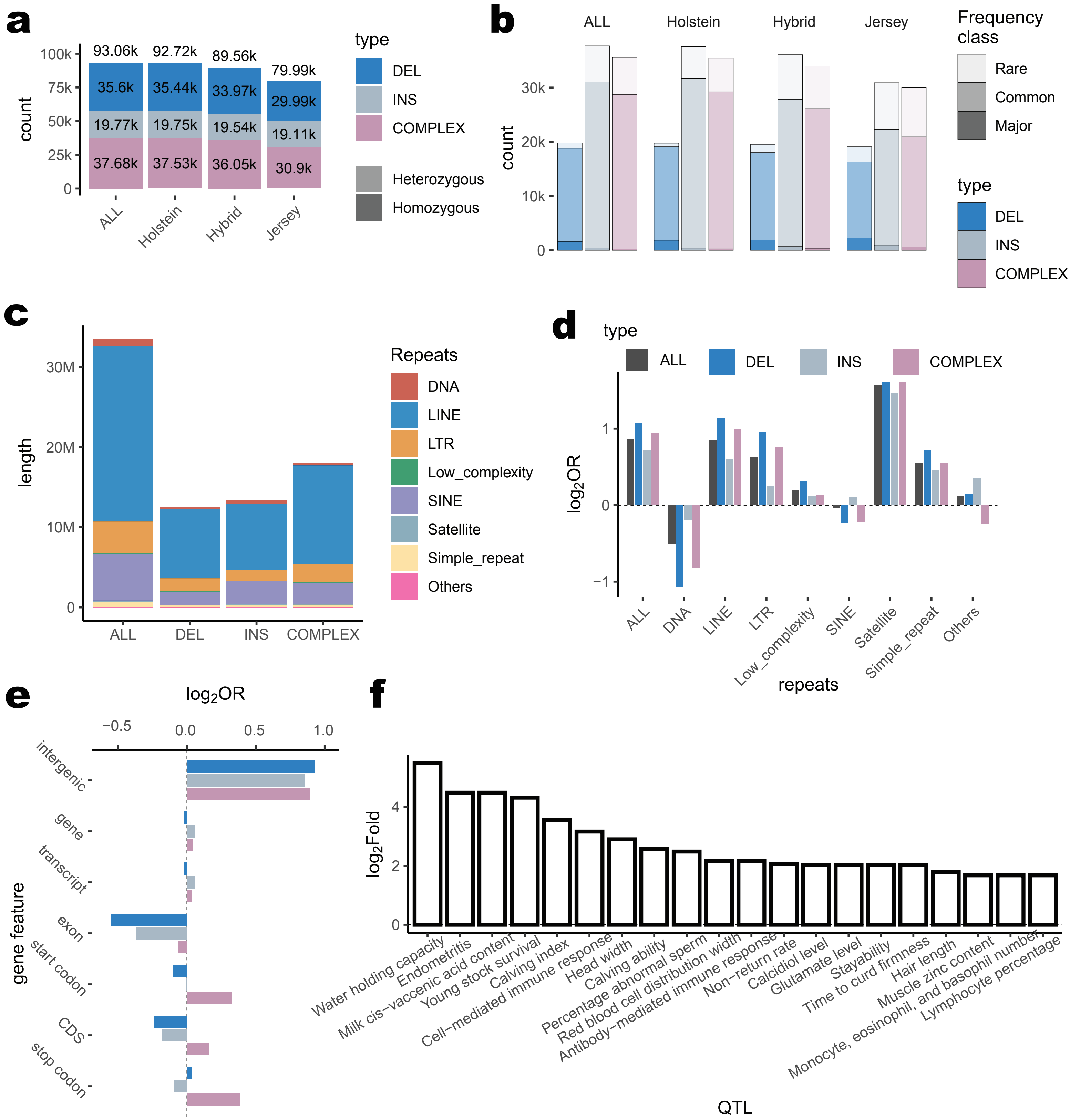

Figure S1. Variation distribution of pangenome SVs from 1571 cattle.
(a) SV count across each group. **(b)** Distributions of SV counts at different frequencies. Based on allele frequency, three SV types were categorized into the major (AF ≥ 0.95), common (0.95 > AF ≥ 0.05), and rare (AF < 0.05) types. **(c)** Proportion of SVs overlapping with common repeats. **(d)** Enrichments of repeats in SV against the whole genome. The y-axis denotes the odds ratio (OR) for the length of each repeat overlapped with SVs, using the genome total length as background, transformed by $\log_2$. The dashed line is at y=0, which indicates that values above the line show a positive association between SVs and repeats, whereas values below indicate a negative association. **(e)** SV annotation based on protein-coding gene features. The panel shows the Chi-squared test results for enrichments of protein-coding genes overlapped with SVs. **(f)** Enrichment of QTLs overlapping with SVs.

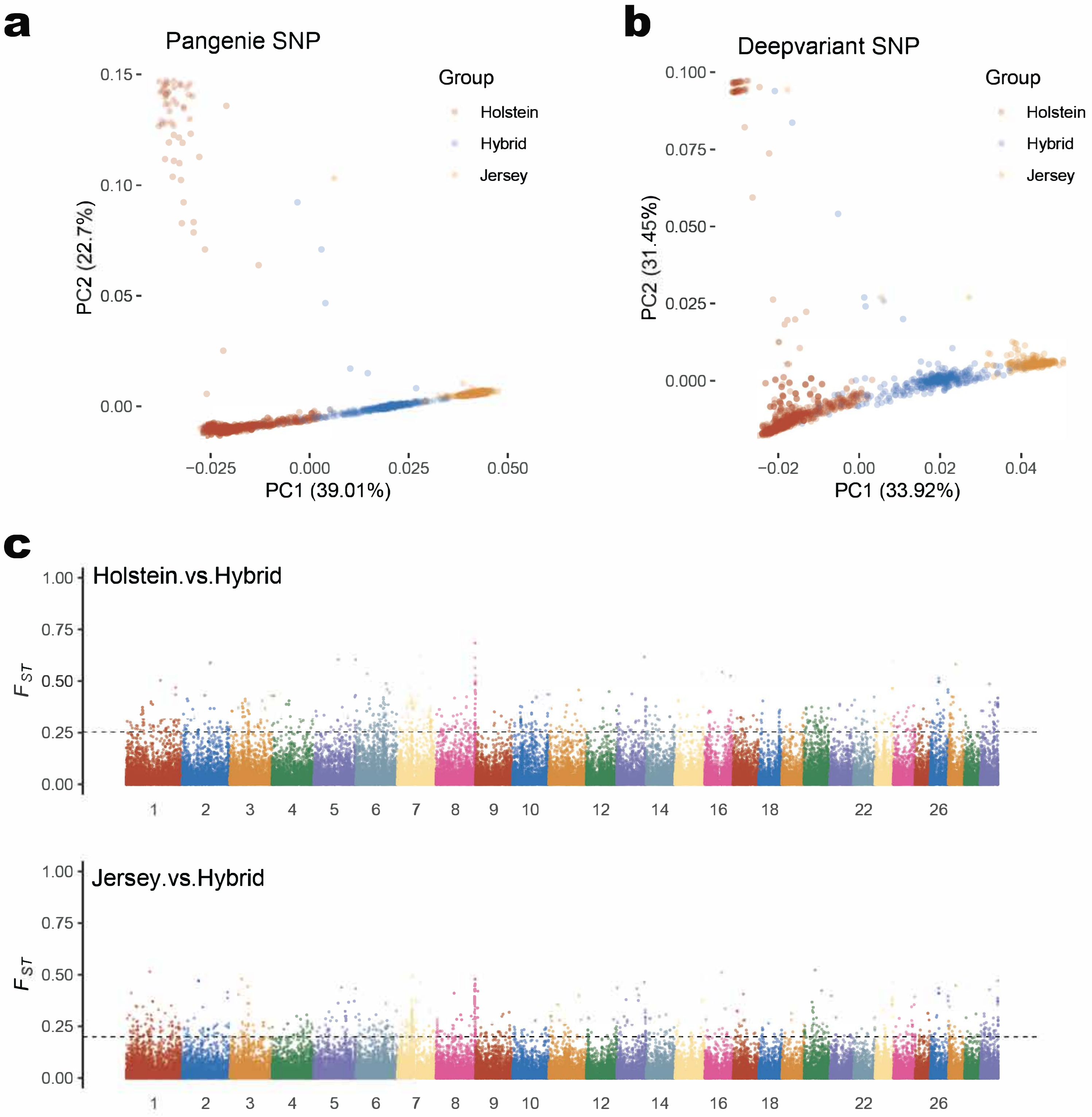

Figure S2. Principal component analysis (PCA) and F_{ST} statistics for 1571 cattle.
(a) PCA for 1571 cattle based on PanGenie SNPs. The explained variation percentages for PC1 and PC2 (in parentheses) were calculated using PLINK2. **(b)** PCA of 1571 cattle based on DeepVariant SNPs. (c) Manhattan plot showing F_{ST} statistics of SVs for Holstein vs. Hybrid, and Hybrid vs. Jersey. SVs above the top 1% threshold lines (red) were considered as candidate selection signals.

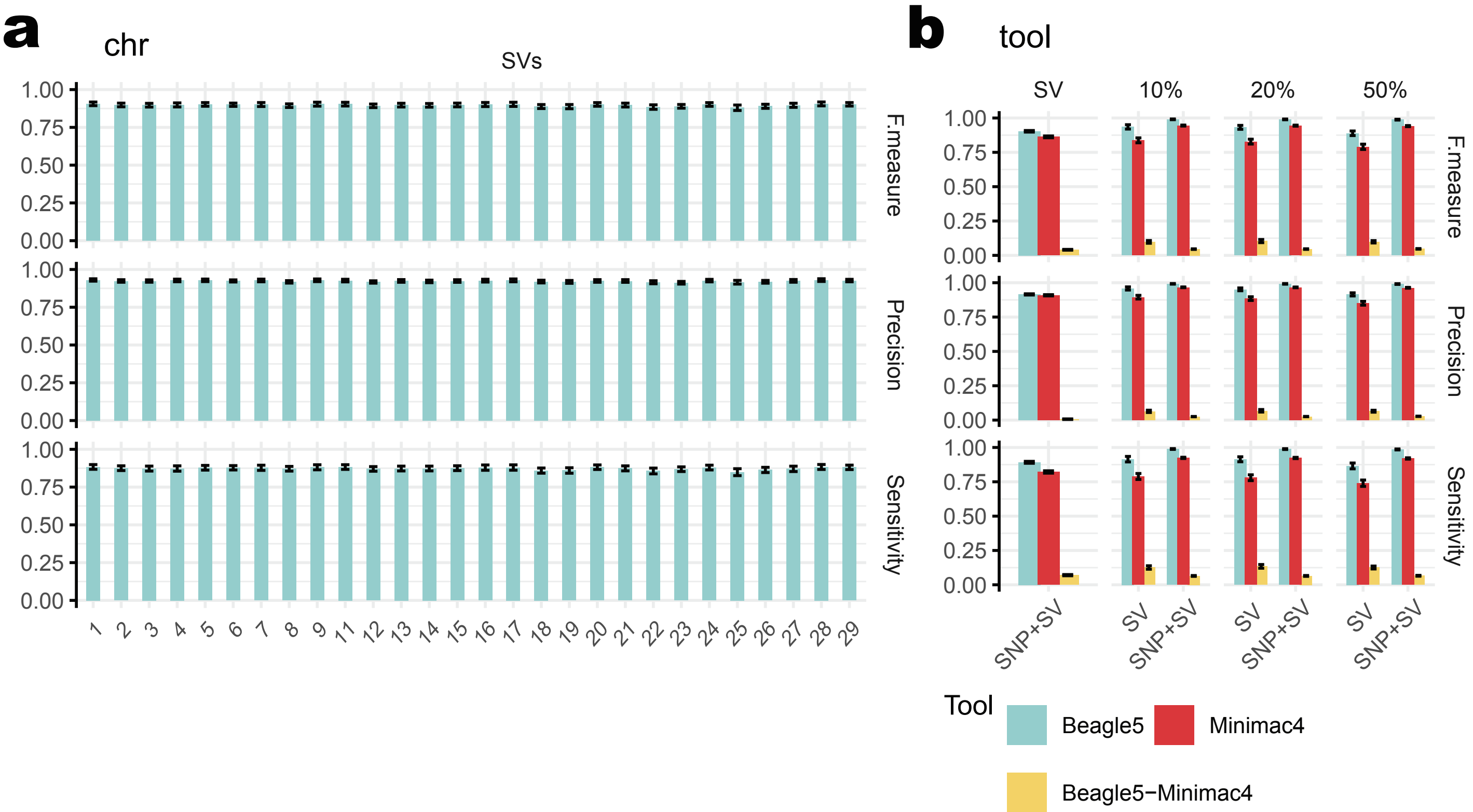

Figure S3. Consistency of imputing SVs using SVs for chromosomes and tools for test samples (n=75). **(a)** Bar plot of Beagle imputation performance for each chromosome of cattle using the SV dataset. **(b)** Bar plot of F1, precision, and sensitivity for Beagle and Minimac using SNP and SV datasets. The top labels (SV, 10%, 20%, 50%) indicate that all SVs, 10% of variants, 20% of variants, and 50% of variants were masked, respectively. Bars represent the mean, and error bars indicate the 95% confidence interval calculated assuming a normal distribution in all panels.

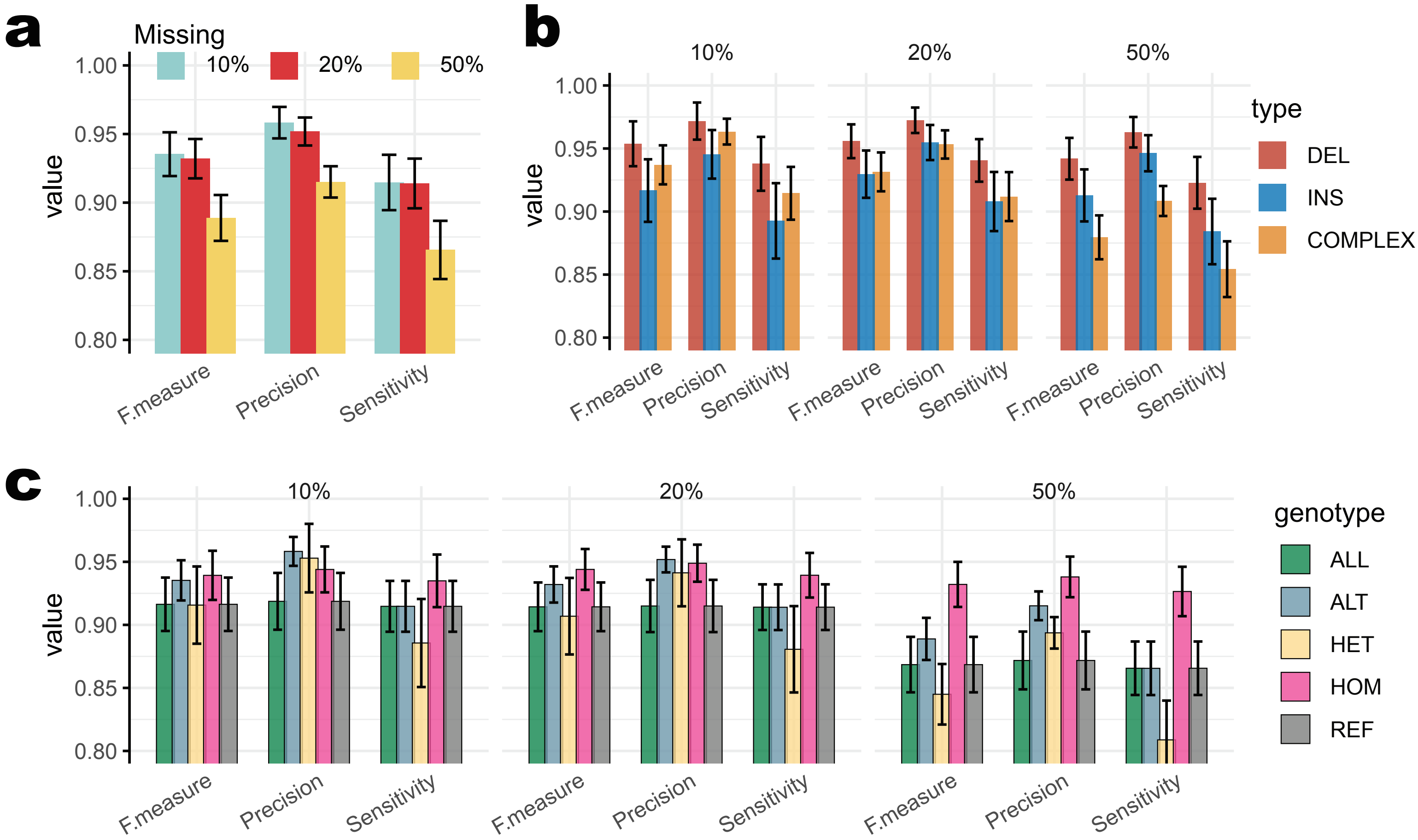

Figure S4. Consistency of imputing SVs using SVs for different masking rates for test samples (n=75). **(a)** Bar plot of imputation performance when SVs were imputed using the PanGenie SV dataset at different masking rates. The top labels 10%, 20%, and 50% indicate that 10%, 20%, and 50% of variants were masked, respectively. **(b)** Imputation performance for different SV types using SV-based imputation. **(c)** Imputation performance for different SV genotypes using SV-based imputation. Bars represent the mean, and error bars indicate the 95% confidence interval calculated assuming a normal distribution in all panels.

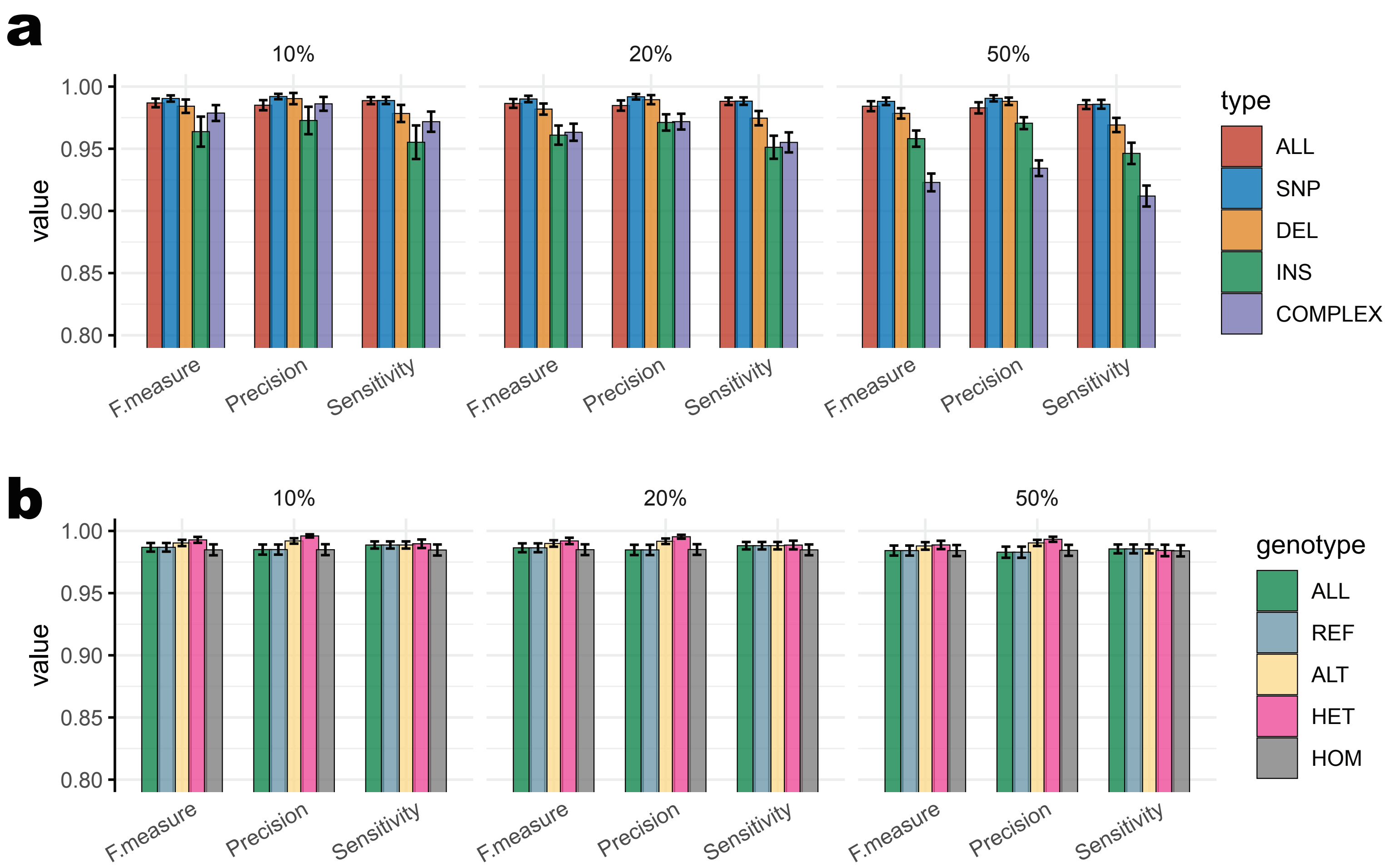

Figure S5. Consistency of imputing SVs using SNPs and SVs for different masking rates for test samples (n=75).
(a) Bar plot of Beagle performance when combining SNP andSV matrices to test different variant types. **(b)** Bar plot of Beagle performance when combining SNP and SV matrix to test different variant genotypes. The top labels (SV, 10%, 20%, 50%) indicate that all SVs, 10% of variants, 20% of variants, and 50% of variants were masked, respectively. Bars represent the mean, and error bars indicate the 95% confidence interval calculated assuming a normal distribution in all panels.

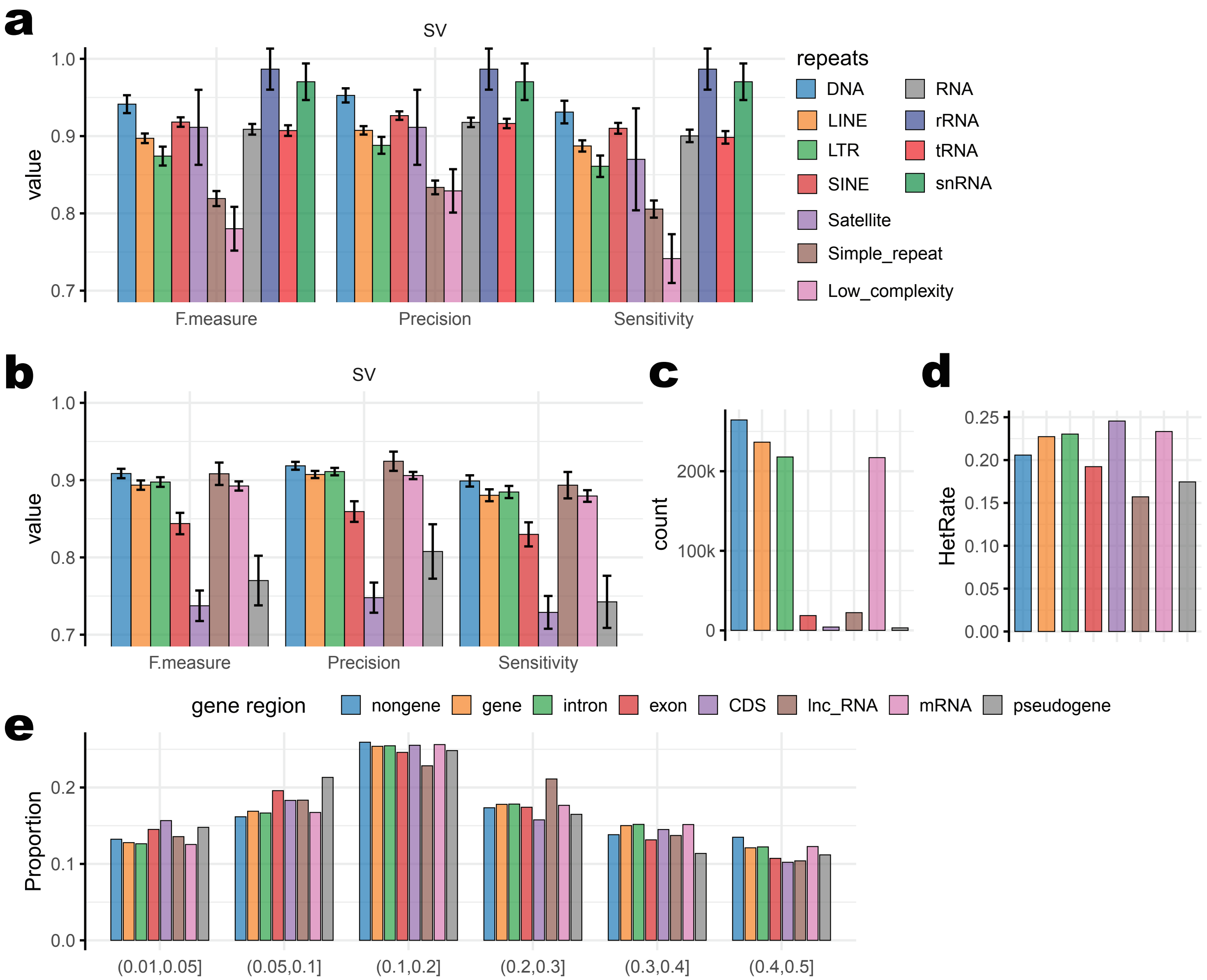

Figure S6. Consistency of imputing SVs using SVs or SNPs for different genome regions for test samples (n=75).
(a) SV imputation performance using SNPs across different repeat regions. **(b)** SV imputation performance using SNPs across different gene regions. **(c)** Variation counts across different gene regions. **(d)** Heterozygous variation rate across different gene regions. **(e)** Proportion of variations in each MAF category across different gene regions. Bars represent the mean, and error bars indicate the 95% confidence interval calculated assuming a normal distribution in all panels.

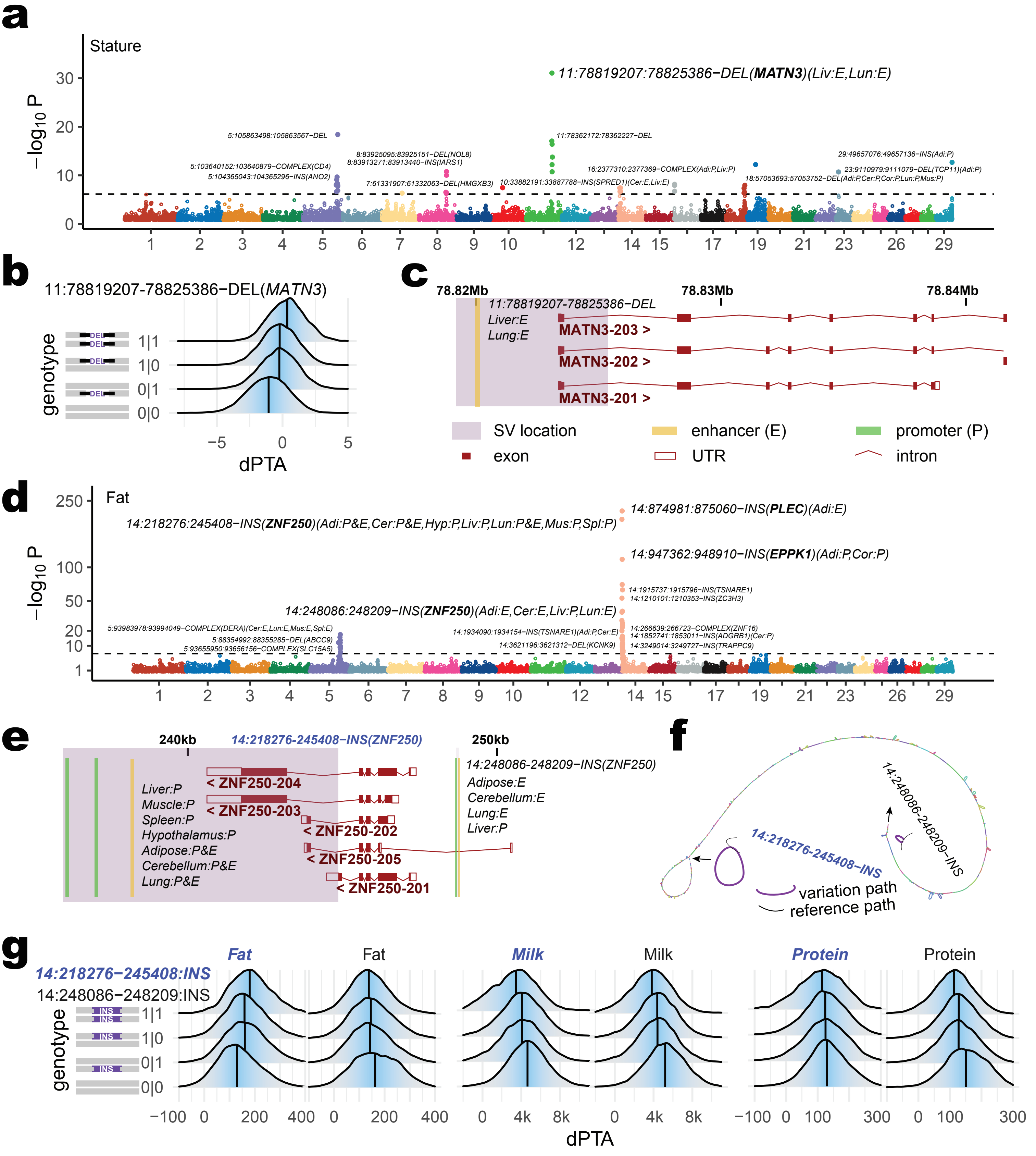

Figure S7. Significant associations of GWAS between imputed SVs and phenotype traits
(a) Manhattan plot of GWAS -$\log_{10}$ *P-values* for imputed SV associated with stature. The x-axis shows the chromosome number and the signal's coordinates, while the y-axis shows -$\log_{10}$ *P-values* for SV-trait associations. The dashed line shows significance thresholds for SVs, and selected significant SVs are labeled. **(b)** The distributions of stature dPTA values for different significant SV genotypes. The X-axis represents dPTA values, with vertical lines showing the mean values. **(c)** Genomic visualization for a significant SV associated with stature, with the SV highlighted in dark pink. **(d)** Manhattan plot of GWAS -$\log_{10}$ *P-values* for imputed SV associated with fat. **(e)** Genomic visualization of a significant SV for fat. **(f)** BandageNG plot for the pangenome nodes starting at 11,022,150 and ending at 11,023,405. Two variations, classified by walks against ARS_UCD2.0, are shown in purple. **(g)** The distributions of stature dPTA values for different significant SV genotypes. Traits labeled in blue italics correspond to SVs also labeled in blue italics.

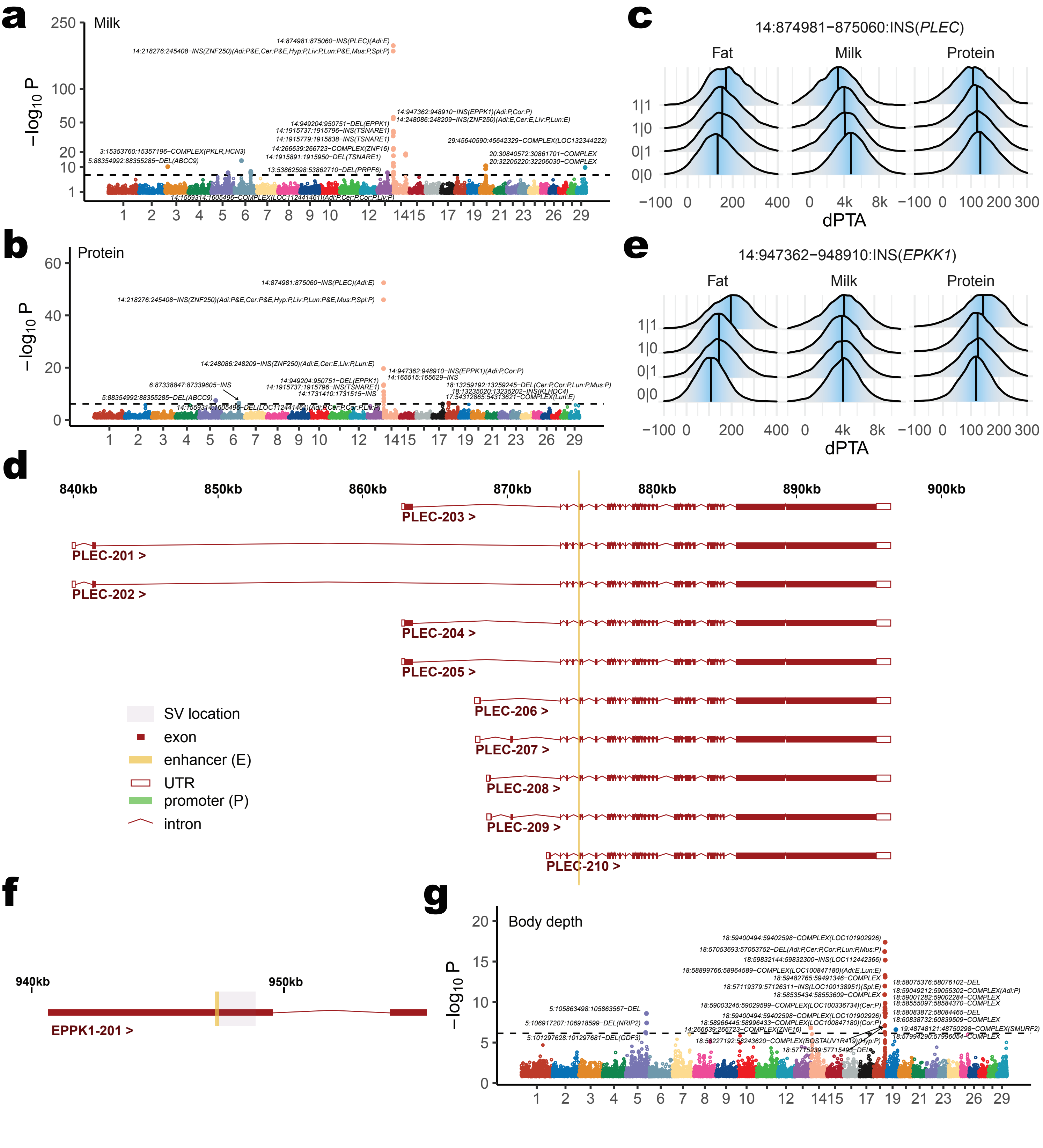

Figure S8. Additional significant GWAS associations between imputed SVs and phenotypic traits in 50,299 samples.
(a) Manhattan plot of GWAS -log$_{10}$ *P-values* for imputed SV associated with milk. The x-axis shows the chromosome number and the signal's coordinates, while the y-axis represents -log$_{10}$ *P-value* for SV-trait associations. The dashed lines indicate significance thresholds, and selected significant SVs are labeled. **(b)** Manhattan plot of GWAS -log$_{10}$ *P-values* for imputed SV associated with protein. **(c)** The distribution of stature dPTA values for different significant SV genotypes. The x-axis represents dPTA values, with vertical lines showing the mean values. **(d)** Genomic visualization for a significant SV. **(e)** The distributions of stature dPTA values for different significant SV genotypes. **(f)** Genomic visualization for another significant SV. **(g)** Manhattan plot of GWAS -log$_{10}$ *P-values* for imputed SV associated with body depth.

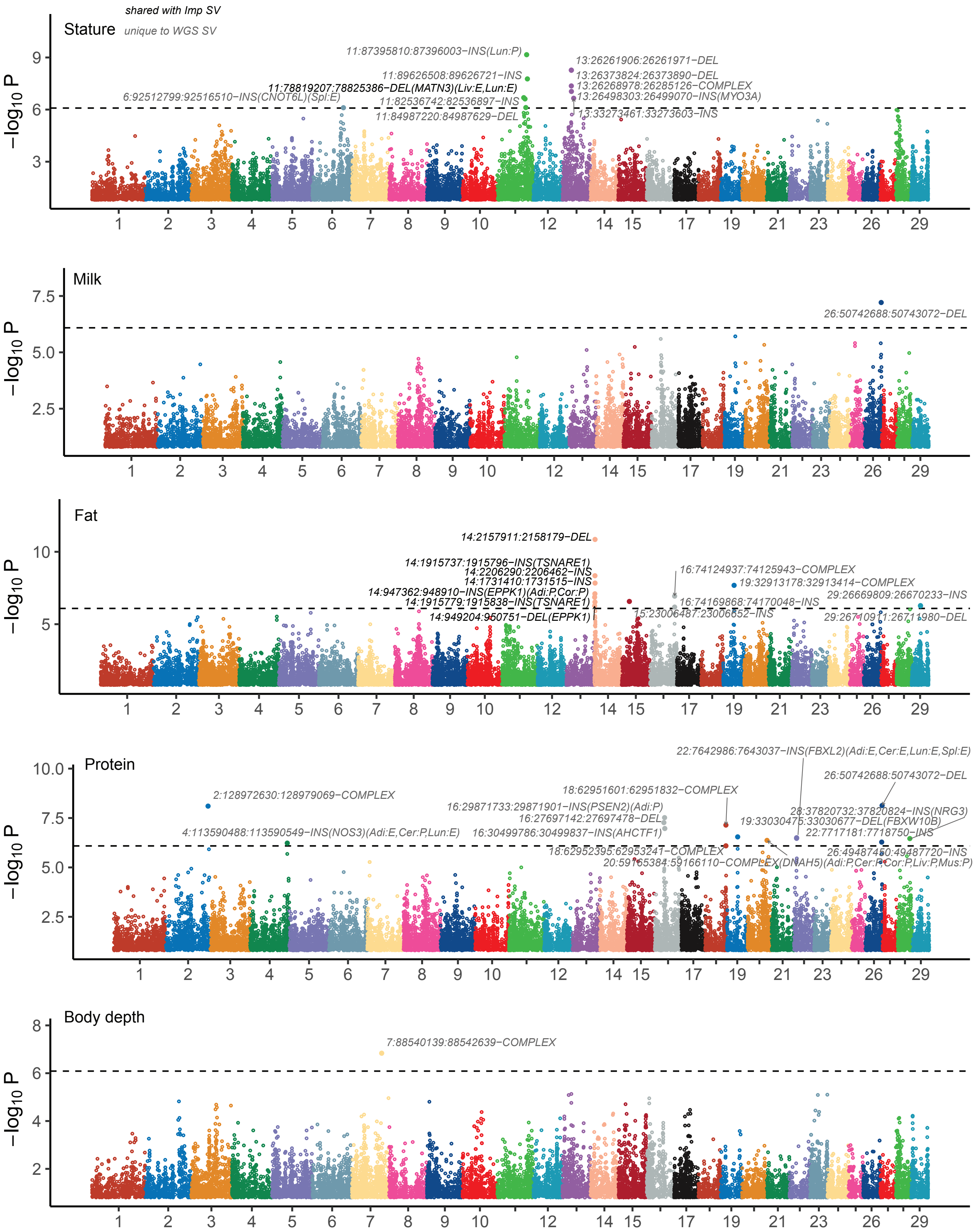

Figure S9. Pangenome SV-based GWAS for 173 WGS cattle data with stature, milk, fat, protein, and body depth traits.
The grey italic texts show unique significant signals from WGS-SV data, while the black regular texts indicate the shared SV with imputed SV GWAS results.

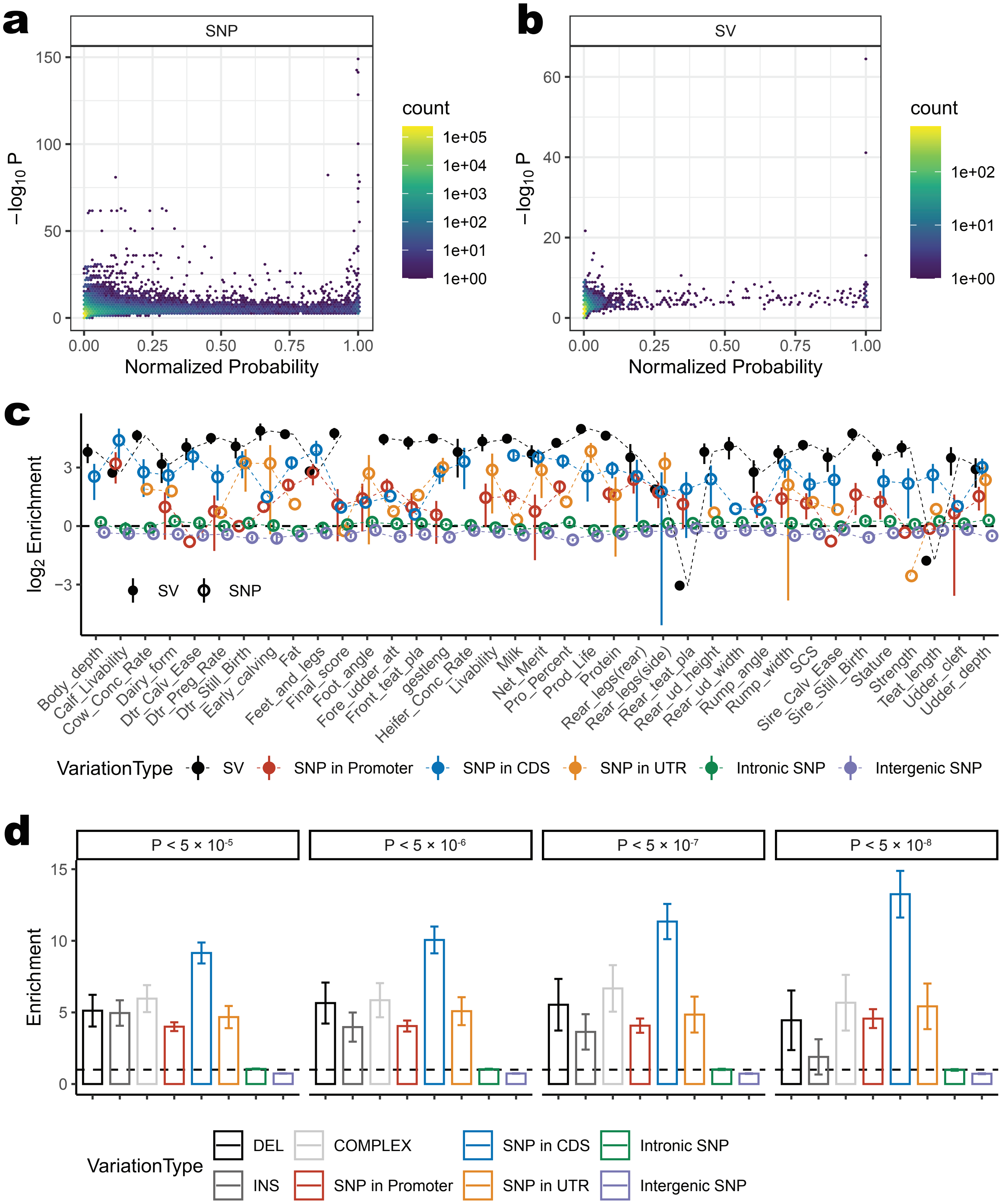

a
SNP
count
1e+05
1e+04
1e+03
1e+02
1e+01
1e+00
−log10 P
Normalized Probability
b
SV
count
1e+02
1e+01
1e+00
−log10 P
Normalized Probability
c
log2 Enrichment
SV
SNP
Body_depth
Calf_Livability
Cow_Conc_Rate
Dairy_form
Dtr_Calv_Ease
Dtr_Preg_Rate
Dtr_Still_Birth
Early_calving
Fat
Feet_and_legs
Final_score
Foot_angle
Fore_udder_att
Front_teat_pla
gestleng
Heifer_Conc_Rate
Livability
Milk
Net_Merit
Pro_Percent
Prod_Life
Protein
Rear_legs(rear)
Rear_legs(side)
Rear_teat_pla
Rear_ud_height
Rear_ud_width
Rump_angle
Rump_width
SCS
Sire_Calv_Ease
Sire_Still_Birth
Stature
Strength
Teat_length
Udder_cleft
Udder_depth
VariationType
SV
SNP in Promoter
SNP in CDS
SNP in UTR
Intronic SNP
Intergenic SNP
d
P < 5 × 10-5
P < 5 × 10-6
P < 5 × 10-7
P < 5 × 10-8
Enrichment
VariationType
DEL
COMPLEX
SNP in CDS
Intronic SNP
INS
SNP in Promoter
SNP in UTR
Intergenic SNP

Figure S10. Enrichment of fine-mapped SNPs and SVs across SV types, functional annotations, and significance thresholds.
(a-b) Distribution of $-\log_{10}$ *P-values* versus normalized posterior inclusion probability (PIP) for fine-mapped SNPs **(a)** and SVs **(b)**, highlighting the relative sparsity of high-confidence SVs. The color scale indicates the density of variants at each probability and significance level. **(c)** Log_2 enrichment of SVs (black circles) and SNPs (open circles) across traits and annotation types for a total of 50,299 cattle. Enrichment was calculated relative to the genome-wide background. SNPs are grouped by annotation: promoter (red), CDS (blue), UTR (orange), intronic (green), and intergenic (purple). Circles indicate mean enrichment estimates, and error bars indicate the 95% confidence interval calculated assuming a normal distribution. Circles indicate mean enrichment estimates. MPH enrichment represents the relative contribution of a given variant category to mid-parent heterosis compared with the genome-wide expectation. An enrichment value greater than 1 indicates that this category contributes disproportionately to MPH. Error bars denote the standard error (SE) of the enrichment estimate, reflecting uncertainty due to sampling and genomic variation. **(d)** Enrichment of variant categories under increasing GWAS significance thresholds ($P < 5 \times 10^{-5}$ to $P < 5 \times 10^{-8}$) for a total of 50,299 cattle. SVs are shown by subtype: DEL (black), INS (pink), and COMPLEX (gray). SNPs are subdivided by functional annotation. CDS SNPs consistently show the highest enrichment among SNP categories, while COMPLEX SVs achieve the greatest enrichment among SV types. Bars represent the mean, and error bars indicate the 95% confidence interval calculated assuming a normal distribution in all panels. Genomic enrichment represents the relative contribution of a given variant category to the overall genetic effect compared with the genome-wide expectation. An enrichment value greater than 1 indicates that this category is overrepresented in explaining genetic effects. SE error bars of the enrichment was estimated by resampling genomic segments using a block jackknife approach, which accounts for linkage disequilibrium and uneven variant distribution across the genome. In panels (c) and (d), the points and bars represent model-derived enrichment estimates rather than sample means, and the error bars represent uncertainty in those estimates. Consequently, no underlying observation-level data distributions exist for overlay.
