# Supplementary material for: Pangenome-based structural variant imputation enables large-scale genotype-phenotype studies in dairy cattle
Source: Nat Commun. 2026 Jul 3;17:8281. doi: 10.1038/s41467-026-75219-x (PMC13469606; doi:10.1038/s41467-026-75219-x)
Supplement: Supplementary file 2 [file 41467_2026_75219_MOESM2_ESM.pdf]

Description of Additional Supplementary Files

Supplementary Data 1

Description: Sample list for public WGS data.

Supplementary Data 2

Description: Pangenome SV list of 1571 public cattle WGS samples.

Supplementary Data 3

Description: Summary statistics of SV genotypes.

Supplementary Data 4

Description: SV genotype statistics for each sample.

Supplementary Data 5

Description: Summary statistics of SV frequency categories.

Supplementary Data 6

Description: SV frequency statistics for each type.

Supplementary Data 7

Description: Summary statistics of SVs overlapping repeat regions.

Supplementary Data 8

Description: List of SVs overlapping genes.

Supplementary Data 9

Description: Summary statistics of SVs overlapping gene biotypes.

Supplementary Data 10

Description: Summary statistics of SVs overlapping gene features.

Supplementary Data 11

Description: Summary statistics of SVs overlapping chromatin states.

Supplementary Data 12

Description: Summary statistics of SVs overlapping QTLs.

Supplementary Data 13

Description: List of high-F_{ST} SVs.

Supplementary Data 14

Description: List of genes associated with high-FST SVs.

Supplementary Data 15

Description: SV imputation consistency rate by chromosome.

Supplementary Data 16

Description: SV imputation consistency rate using Beagle and Minimac.

Supplementary Data 17

Description: SV imputation consistency rate by SV type.

Supplementary Data 18

Description: SV imputation consistency rate by SV allele type.

Supplementary Data 19

Description: SV imputation consistency rate across variation datasets.

Supplementary Data 20

Description: SV imputation consistency rate for SNP and SV allele types.

Supplementary Data 21

Description: SV imputation consistency rate by group.

Supplementary Data 22

Description: SV imputation consistency rate in repeat regions.

Supplementary Data 23

Description: SV imputation consistency rate in gene feature regions.

Supplementary Data 24

Description: Heterozygous and homozygous SNP counts in gene feature regions.

Supplementary Data 25

Description: SV imputation consistency rate across different levels of SNPs and INDELs.

Supplementary Data 26

Description: SV imputation consistency for PanGenie and GATK SNPs in a validation WGS data.

Supplementary Data 27

Description: Beagle DR^2 for each SV site from PanGenie and GATK SNPs in a validation WGS data.

Supplementary Data 28

Description: Proposition of SV with DR2 larger than 0.8 for each SNP density category

Supplementary Data 29

Description: List of imputed SVs with Beagle DR^2 and alternative allele frequency.

Supplementary Data 30

Description: Trait list for Imputed SV-based GWAS

Supplementary Data 31

Description: Significant GWAS signals between imputed SVs and phenotype traits.

Supplementary Data 32

Description: List of significant SVs overlapped by genes.

Supplementary Data 33

Description: List of significant SVs overlapped by enhancers or promoters.

Supplementary Data 34

Description: Number of significant SVs for each trait.

Supplementary Data 35

Description: Number of significant traits for each SV.

Supplementary Data 36

Description: Average dPTA values for each SV genotype with significant associations.

Supplementary Data 37

Description: Statistics of shared and unique SVs for the 5 traits with significant associations.

Supplementary Data 38

Description: Statistics of shared and unique SVs for an independent dataset.

Supplementary Data 39

Description: List of fine-mapping SNPs and SVs.

Supplementary Data 40

Description: List of fine-mapping SVs with annotation ID.

Supplementary Data 41

Description: MPH analysis for each trait.

Supplementary Data 42

Description: Small-effect enrichment of SV and SNP for each trait.

Supplementary Data 43

Description: Small-effect enrichment of SV and SNP for each trait group.

Supplementary Data 44

Description: Large-effect enrichment of SV and SNP for each trait group.
