# Supplementary material for: Pangenome-based structural variant imputation enables large-scale genotype-phenotype studies in dairy cattle
Source: Nat Commun. 2026 Jul 3;17:8281. doi: 10.1038/s41467-026-75219-x (PMC13469606; doi:10.1038/s41467-026-75219-x)
Supplement: Supplementary file 4 [file 41467_2026_75219_MOESM48_ESM.pdf]

nature portfolio

Peer Review File

Pangenome-based structural variant imputation enables large-scale genotype-phenotype studies in dairy cattle

Corresponding Author: Dr George Liu

This file contains all reviewer reports in order by version, followed by all author rebuttals in order by version.

Version 0:

Reviewer comments:

Reviewer #1

(Remarks to the Author)
In this study, Yang et al. contribute to the growing body of work advocating for the use of pangenomes to accurately genotype structural variants (SVs) for the purpose of their inclusion in GWAS studies. The manuscript appropriately emphasizes the need to apply the well documented methods for imputation of SNVs to SVs. Few studies, even in humans, have used pangenome references to increase the number of detected SVs and show the utility of their imputation for GWAS advances. While the authors consistently apply previously published methods, the emphasis on "customized" pangenome builds to improve SV discovery and genotyping was covered in a previous manuscript by these same authors. Therefore, the manuscript would benefit from simplification, particularly through the removal or relegation of non-essential analyses of pangenome references that are compositionally best. There is considerable overlap on this concept of one pangenome is better than another for SV genotyping. Therefore, I suggest streamlining this study with a rewrite to focus only on the imputation narrative and the demonstration of it's effectiveness of imputed SVs for GWAS studies. Moreover, the sections on SNV calling using pangenomes are less impactful, given that this is not a major advantage of pangenome references compared to linear T2T-like genomes. A concise summary of the SNV results in the main text, with detailed results moved to the supplement, would improve focus. That said, this study does offer valuable insights, particularly regarding the performance of imputed SVs, even if the result is disappointing from a GWAS discovery perspective.
Comments by section:
Introduction
I see on lines 59-60, 66 a long dash that is typical of ChatGPT output. Is this correct? If not, what is the dash denoting in sentence structure?
Yang et al. 2025 under review needs to provide a real citation, e.g. bioRxiv.
The last part of the introduction starting at line 93-100 is saturated with numeric results from the study. This should be generalized in the set up for the results section of the paper.
In summary, I recommend a partial rewrite of the introduction that more concisely frames the study within the context of prior work to impute SVs from various references sources and sequence technologies; linear vs pangenome with long read vs short read data. This should include a concise summary of a few relevant imputation studies in other species. This would provide a stronger conceptual foundation and better contextualize the goals and significance of the current study.

Results

Line 110 used the word COMPLEX but I don't see it defined previously in the background section. Define it in its first use.

Line 123 states most SVs overlap repetitive elements. This should be followed with a clarifier that this observation is mostly insertions not deletions based on my experience and the literature.

Line 132. Again acronyms not that commonly used need a definition when first used, e.g. IGC.

Line 137 should be 37,684 not 376,84.

Line 145 why filter at <1Mb. Some explanation would be helpful.

Line 172 what is a 10-fold validation approach. I can make some assumptions, but this is not defined for the general reader.

Line 227. The lowest F1 score within genic regions requires more explanation than lower SV frequency and higher heterozygosity. Genic regions don't show higher heterozygosity compared to non-genic regions. This finding requires a bit more explanation.

Line 273. In this PanGenie performance why did only the RNA SNPs show an F1 score lower than 0.80?

Line 310-314. The detection of 1,225 SV associations seems high for 38 traits. Did the SNP GWAS of this same data show significant hits in these same SV regions? The question is did SVs find new associations not found in the SNP GWAS?

Line 318. Functions is misspelled.

Line 382-384. This statement suggests the inclusion of SVs explains little of the missing genetic variance (increase of 0.11%) beyond what is achievable with SNPs. Therefore, a key reason for implementing SV imputation is that their inclusion in genome wide selection indices would improve our accounting of GWAS signals but this study shows very little improvement. A short explanation would be nice here for this perhaps unexpected outcome.

Discussion.
The discussion revisits themes previously explored in human studies where data coverage was admittedly limited on the use of pangenomes for SV imputation. The key novelty here lies in extending these approaches to cattle with a focus on GWAS improvements. I suggest that in the second paragraph (lines 452–458), the authors provide a sharper contrast to the UK Biobank study on SV use in GWAS, as this comparison would help clarify both the similarities and the distinct contributions of the present work.
I commend the authors for demonstrating, for the first time, the use of pangenome references beyond humans to generate imputation data for both SNVs and SVs, thereby evaluating their potential to enhance GWAS discovery. However, the lack of additional explained genetic variance beyond SNPs highlights the need to reconsider the extent to which SV data can realistically improve selection indices for economically important traits in food-producing animals. I encourage the authors to include forward-looking suggestions in the discussion on how this challenge might be addressed in future work.
I did not identify any major methodological flaws in the study design or in the use of the algorithms applied. That said, the manuscript would benefit from clearer messaging and a more explicit acknowledgment that many of the results are consistent with earlier reports on pangenome-based genotyping in other species. The principal significance of this work lies in its sharing of imputation results across diverse cattle datasets, which represents a valuable resource for the community. Importantly, this study also provides insights that can guide the computational design of future projects, particularly those focused on improving the efficiency of SV imputation across large whole-genome sequencing cohorts.

Reviewer #2

(Remarks to the Author)
The authors conducted an interesting pangenome-based genome-wide study on structural variation genotyping, imputation, and genotype-phenotype association. Given the importance of the work, it is important to enable readers to reproduce the results of the analyses. Detailed methods are appreciated, but they are not enough. Therefore, the authors must prepare a GitHub (or similar) repository with all the steps run to reproduce all the analyses exactly. I also suggest uploading intermediate inputs to facilitate testing and reproducibility.

The authors group the variants by INSERTION, DELETION, and COMPLEX. What about inversions?

Line 65-66: Pangenomes are not pangenome graphs. Pangenomes are sets of sequences, while pangenome graphs are one of the possible representations to represent pangenomes.

178-179: What limits the imputation accuracy to go beyond 0.95? Limited training set (the pangenome is not yet sufficiently representative)? Rare variants? Methodological limitations?

188-189: Why are COMPLEX variants easier to genotype than INSERTIONS? Why are INSERTIONS the hardest variants to genotype?

249-251: Is there a way to determine which are the most informative regions or characteristics that influence imputation accuracy?

Reviewer #3

(Remarks to the Author)
This article is well-written, original and rich in content, and therefore very interesting. However, I have a number of remarks and questions.
1. The panel is Holstein only. While this is appropriate for Holsteins, its use for Jerseys is questionable. SV that are common to both breeds can certainly be imputed, but not those that are rare in Holsteins or specific to Jerseys. In different places, it is claimed that the panel works for both breeds, but without any formal validation using Jersey data, this is an excessive claim. I would suggest limiting these claims to Holsteins and discussing the likely limitations in Jerseys, or simply dropping all

Jersey results. You wrote, 'Our findings underscore the advantages of using breed-specific pangenome references for SV genotyping and imputation', but a multi-breed pangenome would probably be a better option for multi-breed targets.
2. The panel is already quite good but it is still limited to 40 haplotypes. Therefore only SVs present in these 40 haplotypes can be imputed. It would be worth to be discussed.
3. The GWAS thresholds are low (-log(p)=6.1). The SVs are indeed compared with all variants, including SNPs, and the total number of tests to consider is therefore much higher than the number of SVs. Consider, for instance, an SV with -log(p) = 6.5 that is close to a SNP with -log(p) = 10. This SV would not be a relevant candidate variant.
4. Imputation with SNP variants raises the issue of variants being included in deletions. It seems that the authors included all SNPs for imputation of deletions. I have a feeling that some results for deletions could be unpredictable or false. I would strongly recommend excluding these SNPs present in deleted sequences from the SV imputation process.
5. Could you please characterise the complex variations? I would guess that they comprise both a deletion and an insertion, i.e. they are big 'substitutions'. Could you confirm this? How did you handle insertions with a few bases deleted or deletions with a few bases inserted, which are very common?
6. The effect of MATL3 on stature has already been published by Sorin et al.: https://doi.org/10.1186/s12711-025-01012-x. Please cite this reference and adjust your text.Did you check the F1/genotype concordance of genotyping SVs from PanGenie with ones called using HiFi data?
7. Perhaps we could include a discussion about the computational resources required to genotype the SVs from 1,000 animals using PanGenie?

Other remarks:
137: Fix typo.
214–16: You state that the results for Jersey are better than those for hybrids. This is not logical given the nature of the reference panel. Is this due to the higher number of homozygous SVs?
238, 585: Please clarify the number of SNPs for RNA. You mentioned 1,451,919, but it seems that only a small proportion were kept.
245–246: Can you explain why including indels decreases accuracy?
257: Replace "an MAF" with "a MAF".
302: Remove 'successful' as there was no validation of the Jersey results.
309: Net merit is a combination of many traits. I suggest removing this trait.
318: Fix the typo in functions.
678: Milk yield and fat yield are not uncorrelated; the correlation is around 0.6 or 0.7. It would be better to say that highly correlated traits were avoided.
567: I do not understand Fst in '(1 FST 0%, 20%, and 50%)'.
613: Please provide the minimum and mean (or median) number of daughters per bull.
667: How did you treat weights when R^2 was close to 1? Did you bound reliability to avoid very high weights?

Version 1:

Reviewer comments:

Reviewer #1

(Remarks to the Author)
The responses to reviewers critiques were acceptable in most cases.

Reviewer #2

(Remarks to the Author)
Authors addressed all my concerns, except the reproducibility one. They describe a promise to create a detailed GitHub repository, which would be very appreciated, but it is something that I cannot review because it does not yet exist. Authors should make the GitHub repository available before acceptance.

Reviewer #3

(Remarks to the Author)
The manuscript has undergone significant revision, for which I thank the authors.
However, I still have some remarks that need to be addressed:

Regarding the imputation of Jersey, you have only added a few lines of discussion but have maintained most of your results. I am not at all confident about the Jersey results because we know that it does not work well with SNPs. Only frequent SV shared across breeds can be imputed. If you really want to maintain these results, you should mention from the beginning that it is a (unvalidated) test.

Lines 510-515, you stated in your rebuttal letter that Sorin et al. (https://doi.org/10.1186/s12711-025-01012-x), who demonstrated the impact of MATN3 deletion on stature, are now cited. However, I cannot find this reference in the manuscript. Please add this reference.

Regarding the use of SNPs located in a deletion, I am not at all convinced by your answer. Of course, for SNPs located in deleted sequences, genotypes are missing for homozygous deleted individuals and there is only one allele for heterozygous individuals. It is very likely that they are imputed incorrectly in the imputation process and this can provide unpredictable results. I acknowledge that removing them would have implied to recompute all imputations and GWAS from scratch, but at least discuss that point in the manuscript.

Abstract: You mention 1,225 SV-trait associations, but it would be more accurate to state that only 32 of these are good candidate variants and the rest are merely associations.

Line 473, the author wrote: 'Consistent with long homozygous haplotypes shaped by intensive selection.' A more likely explanation is that the minor allele frequency (MAF) is quite low, and homozygous variants (mainly with the major allele), are much easier to impute than other genotypes.

463: Fix typo in review.

Version 2:

Reviewer comments:

Reviewer #3

(Remarks to the Author)
My remarks have been appropriately accounted for. I do not have any additional comment.

Open Access This Peer Review File is licensed under a Creative Commons Attribution 4.0 International License, which permits use, sharing, adaptation, distribution and reproduction in any medium or format, as long as you give appropriate credit to the original author(s) and the source, provide a link to the Creative Commons license, and indicate if changes were made.
In cases where reviewers are anonymous, credit should be given to 'Anonymous Referee' and the source.
The images or other third party material in this Peer Review File are included in the article’s Creative Commons license, unless indicated otherwise in a credit line to the material. If material is not included in the article’s Creative Commons license and your intended use is not permitted by statutory regulation or exceeds the permitted use, you will need to obtain permission directly from the copyright holder.
To view a copy of this license, visit https://creativecommons.org/licenses/by/4.0/

REVIEWER COMMENTS

Reviewer #1 (Remarks to the Author):

In this study, Yang et al. contribute to the growing body of work advocating for the use of pangenomes to accurately genotype structural variants (SVs) for the purpose of their inclusion in GWAS studies. The manuscript appropriately emphasizes the need to apply the well documented methods for imputation of SNVs to SVs. Few studies, even in humans, have used pangenome references to increase the number of detected SVs and show the utility of their imputation for GWAS advances. While the authors consistently apply previously published methods, the emphasis on "customized" pangenome builds to improve SV discovery and genotyping was covered in a previous manuscript by these same authors.
Q11. Therefore, the manuscript would benefit from simplification, particularly through the removal or relegation of non-essential analyses of pangenome references that are compositionally best. There is considerable overlap on this concept of one pangenome is better than another for SV genotyping.
AU: We appreciate the reviewer's comment. All sections discussing comparisons among pangenome references or identifying an "optimal" pangenome for SV genotyping have been fully removed from this manuscript. These analyses were part of a separate study (Yang et al., 2025, in press) and were inadvertently overlapping because both manuscripts were reviewed at the same time. Their removal has substantially simplified the manuscript and sharpened the focus on SV imputation and its downstream applications.

Q12. Therefore, I suggest streamlining this study with a rewrite to focus only on the imputation narrative and the demonstration of it's effectiveness of imputed SVs for GWAS studies.
AU: We agree with the reviewer's recommendation to streamline the study around the imputation narrative. In response, we have extensively revised the manuscript so that its central storyline now focuses exclusively on SV imputation and the demonstration of its effectiveness for GWAS. The Introduction and Discussion were rewritten accordingly, all pangenome-comparison benchmarking has been removed, and content related to SNV calling performance has been relocated to the companion manuscript. The present study now follows a cohesive arc, SV imputation, validation, and GWAS utility, and the remaining SNV material is limited to essential context required to interpret the imputation panel and downstream analyses.

Q13. Moreover, the sections on SNV calling using pangenomes are less impactful, given that this is not a major advantage of pangenome references compared to linear T2T-like genomes. A concise summary of the SNV results in the main text, with detailed results moved to the supplement, would improve focus.
AU: We appreciate the reviewer's thoughtful suggestion and wish to clarify the source of possible confusion. Two manuscripts from our group were submitted and reviewed concurrently: one benchmarking pangenome reference performance (including SNV calling and demonstrating that H20D performs best) and this manuscript, which focuses on SV imputation and GWAS. These were originally part of a single *Nature Genetics* submission and were later separated, which likely led to overlap being perceived in the review process. Following the separation, all

analyses comparing or validating pangenome references were removed from this manuscript and placed solely in the companion study (Yang et al., 2025, in press). The SNV section that remains in the current manuscript is not a benchmarking component; rather, it provides the necessary genomic context for interpreting the HolPIP panel and the integration of SVs with SNP-based GWAS. This concise summary serves critical functions, anchoring imputed SVs within the broader genomic landscape, supporting interpretation of SV–SNP integration, and maintaining narrative continuity. Because of its importance and the restructuring already completed, we respectfully prefer not to further condense this section (Figure 3a–b) or move it entirely to the Supplement.

That said, this study does offer valuable insights, particularly regarding the performance of imputed SVs, even if the result is disappointing from a GWAS discovery perspective.

Comments by section:

Introduction

Q14. I see on lines 59-60, 66 a long dash that is typical of ChatGPT output. Is this correct? If not, what is the dash denoting in sentence structure?

AU: Thank you for noting this. The long dashes on the indicated lines were unintentional formatting artifacts. We have revised the text to use standard punctuation and ensured consistency throughout the manuscript.

Q15. Yang et al. 2025 under review needs to provide a real citation, e.g. bioRxiv.

AU: It should be published by then.

Q16. The last part of the introduction starting at line 93-100 is saturated with numeric results from the study. This should be generalized in the set up for the results section of the paper.

AU: We removed numbers and updated to "Here, we used a combination of a Holstein-specific pangenome and graph-based genotyping approaches to comprehensively characterize both single-nucleotide and structural variation from short-read sequencing data in a large cattle population. We then developed a pangenome-based reference panel to enable genome-wide imputation of structural variants at population scale. By integrating these data with phenotypes for economically important traits, our subsequent genome-wide analyses identified multiple structural variants associated with variation in growth, production, and other complex traits. Together, these results demonstrate the power of breed-specific pangenomes and structural variant imputation for capturing previously underexplored genetic variation and highlight their potential to enhance genomic selection and accelerate genetic improvement in cattle."

Q17. In summary, I recommend a partial rewrite of the introduction that more concisely frames the study within the context of prior work to impute SVs from various references sources and sequence technologies; linear vs pangenome with long read vs short read data. This should include a concise summary of a few relevant imputation studies in other species. This would provide a stronger conceptual foundation and better contextualize the goals and significance of the current study.

AU: We revised the Introduction to more concisely frame our study within the context of prior work on SV imputation across different reference types and sequencing technologies,

highlighting linear versus pangenome approaches and long-read versus short-read data. On lines 70–78, we included a brief summary of relevant studies in humans (UK Biobank), tomato, rice, and other species, as recommended. These additions provide a stronger conceptual foundation and better contextualize the goals and significance of our work.

Results

Q18. Line 110 used the word COMPLEX but I don't see it defined previously in the background section. Define it in its first use.
AU: On L106, we added '"COMPLEX" refers to variant alleles located within pangenome-graph bubbles containing more than two branches, representing sequence changes of at least two base pairs that cannot be classified simply as DEL or INS'

Q19. Line 123 states most SVs overlap repetitive elements. This should be followed with a clarifier that this observation is mostly insertions not deletions based on my experience and the literature.
AU: We clarified it by adding INS before SVs.

Q20. Line 132. Again acronyms not that commonly used need a definition when first used, e.g. IGC.
AU: We update texts and Tables S9. In all cases, IG was spelled out as Immunoglobulin.

Q21. Line 137 should be 37,684 not 376,84.
AU: Done.

Q22. Line 145 why filter at <1Mb. Some explanation would be helpful.
AU: We filtered QTLs to < 1 Mb because very large intervals are low-resolution and span many genes, making overlaps with SVs uninformative. Sub-megabase QTLs from Animal QTLdb provide higher resolution and more meaningful functional comparisons with SVs.

Q23. Line 172 what is a 10-fold validation approach. I can make some assumptions, but this is not defined for the general reader.
AU: A 10-fold cross-validation approach means that the dataset was randomly divided into 10 equal subsets ("folds"). In each round, nine folds were used to build the imputation model, and the remaining one fold was held out for testing. This process was repeated 10 times, with each fold serving once as the test set. The final accuracy is the average across all 10 rounds, providing a robust and unbiased estimate of imputation performance. On L174, we added ", in which the 750 Holsteins were randomly divided into ten equal subsets and imputation accuracy was evaluated by iteratively training on nine subsets and testing on the remaining one."

Q24. Line 227. The lowest F1 score within genic regions requires more explanation than lower SV frequency and higher heterozygosity. Genic regions don't show higher heterozygosity compared to non-genic regions. This finding requires a bit more explanation.
AU: Thank you for this insightful comment. We agree that the lower F1 score in coding sequences (CDS) requires further clarification. CDS regions contain fewer SVs overall and tend

to harbor a higher proportion of functionally constrained and rare variants due to strong purifying selection. These properties make SVs in CDS more difficult to genotype accurately because (i) the reduced number of SV events limits statistical support for model training, and (ii) functionally important variants often occur in sequence contexts, such as highly conserved, GC-rich, or repetitive micro-motifs, that complicate read alignment and SV breakpoint resolution. Thus, the lower F1 score in CDS is more likely driven by biological constraint and technical alignment challenges, rather than by differences in overall heterozygosity or MAF distribution across genic and non-genic regions. On L232, we modified to "This likely reflects the strong functional constraint in CDS, which results in fewer SVs and a higher proportion of rare, conserved variants whose breakpoints occur in sequence contexts that are more difficult to align and resolve. These biological and technical factors, rather than differences in MAF distribution, contribute to reduced genotyping performance in CDS."

Q25. Line 273. In this PanGenie performance why did only the RNA SNPs show an F1 score lower than 0.80?

AU: The slightly lower F1 score for RNA SNPs likely reflects the inherent challenges of genotyping variants within transcribed regions. RNA SNPs often fall in splicing junctions, UTRs, or highly expressed and structurally dynamic regions, which can introduce alignment ambiguity and increase mapping errors. These features make accurate imputation more difficult compared to SNPs in more stable genomic contexts. As a result, RNA SNPs show a modest reduction in F1 (0.79), while all other SNP categories, located in regions with fewer transcription-related complexities, achieve F1 scores above 0.80.

Q26. Line 310-314. The detection of 1,225 SV associations seems high for 38 traits. Did the SNP GWAS of this same data show significant hits in these same SV regions? The question is did SVs find new associations not found in the SNP GWAS?

AU: Thank you for this comment. Because the SVs in our study were imputed from SNPs, most SV–trait associations correspond to loci already detected in the SNP-based GWAS, and we therefore did not observe many entirely new signals. The SV-GWAS mainly refined or complemented existing SNP associations by highlighting structural variants that may underlie those signals. We anticipate that SVs directly predicted from matched whole-genome sequencing data, rather than imputed, would capture additional variation and are likely to reveal more novel trait-associated loci.

Q27. Line 318. Functions is misspelled.

AU: Corrected.

Q28. Line 382-384. This statement suggests the inclusion of SVs explains little of the missing genetic variance (increase of 0.11%) beyond what is achievable with SNPs. Therefore, a key reason for implementing SV imputation is that their inclusion in genome wide selection indices would improve our accounting of GWAS signals but this study shows very little improvement. A short explanation would be nice here for this perhaps unexpected outcome.

AU: On L400, we added "A likely explanation for the minimal gain is that most large-effect SVs are already in strong linkage disequilibrium with nearby SNPs, so their effects are largely captured by the SNP panel. As a result, adding imputed SVs introduces limited new information

for genomic prediction. Imputation inaccuracies and the generally low allele frequencies of many SVs also reduce their contribution to explained variance. Even so, imputed SVs may still help clarify or fine-map association signals by representing causal variation that nearby SNPs only tag indirectly. It is also possible that using SV genotypes directly obtained from matched long- or short-read sequencing, rather than relying on SNP-based imputation, would provide more complete and accurate SV representations and could therefore uncover additional signals and yield greater improvements in genomic prediction accuracy."

Discussion.

Q29. The discussion revisits themes previously explored in human studies where data coverage was admittedly limited on the use of pangenomes for SV imputation. The key novelty here lies in extending these approaches to cattle with a focus on GWAS improvements. I suggest that in the second paragraph (lines 452–458), the authors provide a sharper contrast to the UK Biobank study on SV use in GWAS, as this comparison would help clarify both the similarities and the distinct contributions of the present work.

AU: We thank the reviewer for this helpful suggestion. We have revised the second paragraph of the Discussion (L465-480) to more clearly contrast our work with the recent UK Biobank SV-GWAS study (Ref. 16). While both studies demonstrate comparable accuracy for population-scale SV imputation and association testing, they differ in biological system, reference framework, and analytical objectives. The human study focused on disease fine-mapping using a large linear-reference-based SV panel, whereas our work applies a pangenome graph framework to livestock and evaluates the contribution of SVs to complex production traits and genomic selection. A unique aspect of our analysis is the explicit comparison of homozygous and heterozygous SV genotypes, with homozygous variants showing higher imputation accuracy (F1 = 0.93) than heterozygous variants (F1 = 0.84), consistent with long homozygous haplotypes shaped by intensive selection. In addition, incorporating SVs also provides a more complete view of the genetic architecture of economically important traits, as SVs show strong functional enrichment despite explaining a modest fraction of additive variance. Together, these findings highlight both the limitations of linear reference genomes and the practical advantages of pangenome graphs for resolving complex structural variation in cattle breeding and genetic improvement.

Key similarities and distinctions

**Feature**	**Human SV-GWAS (Ref. 16, UK Biobank)**	**This study (Holstein Cattle)**
Species/system	Human populations	Livestock (dairy cattle)
Population structure	Large effective population size, short LD (~10kb)	Small effective population size, long LD blocks (~100kb)
Reference framework	Linear reference SV panel from 888 multi-ancestry long-read genomes	Breed-specific pangenome graph (H20D) SV panel from 20 cows
Target cohort	~450k UK Biobank participants	Dairy cattle populations (50k)
Primary focus	Disease association and fine-mapping	Production, fertility, health traits

**Feature**	**Human SV-GWAS (Ref. 16, UK Biobank)**	**This study (Holstein Cattle)**
Downstream emphasis	Post-GWAS prioritization and pQTL	Genomic prediction and breeding relevance

Q30. I commend the authors for demonstrating, for the first time, the use of pangenome references beyond humans to generate imputation data for both SNVs and SVs, thereby evaluating their potential to enhance GWAS discovery. However, the lack of additional explained genetic variance beyond SNPs highlights the need to reconsider the extent to which SV data can realistically improve selection indices for economically important traits in food-producing animals. I encourage the authors to include forward-looking suggestions in the discussion on how this challenge might be addressed in future work.
AU: We appreciate the reviewer's recognition of our demonstration of pangenome-based SV and SNV imputation in livestock. As noted, the limited increase in explained genetic variance beyond SNPs reflects that most imputed SVs are already tagged by nearby SNPs, and their low frequencies or imputation errors further constrain their contribution to genomic prediction. To address this challenge in future work, we suggest three directions: (1) generating SV genotypes directly from matched long- or short-read sequencing to capture rare and complex variants that are poorly represented by SNPs, (2) expanding and diversifying livestock pangenomes to improve representation of breed-specific structural variation, and (3) integrating functional annotation and regulatory information to prioritize SVs most likely to impact trait variation. Together, these strategies could enhance the utility of SVs in genomic selection and improve selection indices for economically important traits.

Q31. I did not identify any major methodological flaws in the study design or in the use of the algorithms applied. That said, the manuscript would benefit from clearer messaging and a more explicit acknowledgment that many of the results are consistent with earlier reports on pangenome-based genotyping in other species. The principal significance of this work lies in its sharing of imputation results across diverse cattle datasets, which represents a valuable resource for the community. Importantly, this study also provides insights that can guide the computational design of future projects, particularly those focused on improving the efficiency of SV imputation across large whole-genome sequencing cohorts.
AU: What our study contributes beyond these earlier efforts is twofold. First, we extend SV imputation to livestock at a scale and breadth not previously achieved, applying pangenome-based SV genotyping across a large collection of cattle datasets. This produces a widely usable imputation resource for the dairy cattle genomics community, filling a gap between the capabilities demonstrated in humans and the much smaller datasets previously available in livestock. Second, we provide a practical, data-driven evaluation of how imputed SVs integrate into genomic studies and breeding-relevant analyses. By explicitly testing whether imputed SVs improve trait-association detection and increase the proportion of explained genetic variance, we offer an empirical assessment of a key assumption in cattle breeding research. Although the improvement in our dataset is modest, this result is itself informative: it clarifies current limitations (e.g., the accuracy of imputed SVs, allele frequency distributions, SNP tagging) and highlights where future efforts, such as deeper sequencing and broader breed representation, are

likely to yield larger gains. Together, these contributions position our work within the broader field of pangenome-based genotyping while extending it into a large-scale livestock setting and offering resources and insights that will help guide future SV-imputation studies in cattle.

Reviewer #2 (Remarks to the Author):

Q32. The authors conducted an interesting pangenome-based genome-wide study on structural variation genotyping, imputation, and genotype-phenotype association. Given the importance of the work, it is important to enable readers to reproduce the results of the analyses. Detailed methods are appreciated, but they are not enough. Therefore, the authors must prepare a GitHub (or similar) repository with all the steps run to reproduce all the analyses exactly. I also suggest uploading intermediate inputs to facilitate testing and reproducibility.

AU: We thank the reviewer for emphasizing the importance of reproducibility. In line with FAIR (Findable, Accessible, Interoperable, Reusable) principles, we will create a publicly accessible GitHub repository containing:

1. All scripts, pipelines, and configuration files used for SV discovery, pangenome construction, imputation, and GWAS analyses.
2. Detailed documentation describing step-by-step procedures, software versions, parameter settings, and dependencies to ensure analyses can be exactly reproduced.
3. Example input files and intermediate outputs where possible, enabling users to test and validate each stage of the workflow.

Due to the size and privacy restrictions of raw WGS data, the repository will provide processed datasets and example subsets sufficient for reproducing key results and figures. This repository will be permanently linked in the Methods section, supporting transparency, reproducibility, and reuse by the research community.

Q33. The authors group the variants by INSERTION, DELETION, and COMPLEX. What about inversions?

AU: We thank the reviewer for the comment. While inversions were detected in the pangenome assemblies, their genotyping and imputation accuracy was lower than that of insertions and deletions. To avoid false positives, we focused on INSERTION, DELETION, and COMPLEX categories. Future work using long-read sequencing or assembly-based genotyping could enable accurate inclusion of inversions in SV imputation and GWAS.

Q34. Line 65-66: Pangenomes are not pangenome graphs. Pangenomes are sets of sequences, while pangenome graphs are one of the possible representations to represent pangenomes.

AU: We agree that “pangenomes” and “pangenome graphs” are distinct concepts: a pangenome is a collection of genome sequences representing the diversity of a species, whereas a pangenome graph is one representation of a pangenome that encodes sequence variation and relationships in a graph structure. To clarify, we have revised the text as follows on L65: "Single-reference genomes fail to capture the full spectrum of SV diversity, fueling the demand for pangenomes, a collection of multiple high-quality assemblies. Pangenome graphs are one approach to represent these sequences in a structure that reflects population-level genetic diversity."

Q35. 178-179: What limits the imputation accuracy to go beyond 0.95? Limited training set (the pangenome is not yet sufficiently representative)? Rare variants? Methodological limitations?

AU: The observed imputation accuracy (average F1 ≈ 0.898) is constrained by multiple biological and technical factors, which together limit performance from exceeding ~0.95 at the current stage.

1. Limited representativeness of the training set (pangenome reference):

Although the pangenome integrates multiple assemblies, it still does not fully capture the haplotype and structural diversity of the global Holstein population. Many structural variants (SVs), especially population-specific or lineage-specific ones, are underrepresented or absent, reducing imputation accuracy for these loci.

2. Low frequency and uneven genomic distribution of SVs:

Compared with SNPs, SVs occur at much lower frequencies and are unevenly distributed across the genome. Rare SVs suffer from sparse linkage disequilibrium (LD) information and limited observations in the reference panel, making accurate imputation inherently more difficult and lowering achievable F1 scores.

3. SV calling and genotyping uncertainty:

Even when using a pangenome as reference, SV detection and genotyping remain less accurate than SNP calling. Tools such as PanGenie can still produce false positives and false negatives due to ambiguous breakpoint definitions, repetitive regions, and complex rearrangements. These upstream inaccuracies propagate into the imputation step and impose an upper bound on achievable accuracy.

4. Methodological limitations of current imputation algorithms:

Most imputation frameworks (e.g., Beagle) are optimized for biallelic, well-phased SNPs. Complex SVs-including multi-allelic variants, nested SVs, or variants with imprecise breakpoints-violate these assumptions and are therefore imputed with reduced accuracy, even when LD information is available.

5. Weaker and shorter-range LD between SVs and surrounding markers:

SVs often exhibit weaker LD with nearby SNPs than SNP-SNP pairs, particularly for insertions and duplications. This limits the predictive power of flanking markers and constrains maximum imputation performance.

In combination, these factors explain why imputation accuracy does not exceed 0.95 in the current dataset. Future improvements are expected from expanding the pangenome reference with more diverse long-read assemblies, improving SV calling and genotyping accuracy, and developing imputation models specifically tailored for complex structural variation. In addition, imputation performance is further constrained by reduced SNP genotyping accuracy within or near SV regions. Structural variants can disrupt local read mapping and alignment, leading to missing or erroneous SNP calls inside SV intervals or at their breakpoints. As a result, the local LD structure between SVs and surrounding SNP markers is degraded, weakening the informative signal required for accurate imputation. This issue is particularly pronounced for insertions, duplications, and complex rearrangements, where short-read-based SNP calling is less reliable. Improving SNP detection and genotyping accuracy in SV-affected regions will therefore be an important factor for further enhancing SV imputation performance.

Q36. 188-189: Why are COMPLEX variants easier to genotype than INSERTIONS? Why are INSERTIONS the hardest variants to genotype?

AU: The observed differences in genotyping accuracy among SV types reflect both technical and biological factors:

1. INSERTIONS are hardest to genotype because they introduce novel sequence not present in the reference genome. Short-read alignment and graph-based imputation rely on existing reference sequences, making it challenging to correctly detect or phase insertions, especially if they are large or rare.
2. COMPLEX variants can be easier to genotype than INSERTIONS because many complex SVs include deletions or other changes that overlap with existing reference sequence. These features provide alignment and marker information that improve genotyping and imputation performance.
3. DELs are most accurately imputed since deletions remove reference bases, which are more easily detected via read-depth or split-read signals, and are generally well tagged by surrounding markers.

Thus, the relative difficulty follows the spectrum: INSERTIONS > COMPLEX > DELETIONS, with marker frequency and availability influencing imputation for complex events.

Q37. 249-251: Is there a way to determine which are the most informative regions or characteristics that influence imputation accuracy?

AU: Yes, the most informative regions or characteristics for imputation accuracy can be assessed by examining marker density, distribution relative to SVs, minor allele frequency, and LD patterns. In our case, although RNA SNPs are abundant, they are often clustered within expressed genes and poorly cover intergenic or regulatory regions where many SVs reside. This uneven genomic distribution reduces their informativeness for imputation. By contrast, HD and LD SNPs are more evenly distributed across the genome, providing better coverage and stronger linkage to SVs, which improves imputation accuracy.

Future work could formalize this assessment by computing per-region imputation F1 or LD-based tagging scores to systematically identify regions or SNP characteristics most predictive of SV genotypes.

Reviewer #3 (Remarks to the Author):

This article is well-written, original and rich in content, and therefore very interesting. However, I have a number of remarks and questions.

Q38. 1. The panel is Holstein only. While this is appropriate for Holsteins, its use for Jerseys is questionable. SV that are common to both breeds can certainly be imputed, but not those that are rare in Holsteins or specific to Jerseys. In different places, it is claimed that the panel works for both breeds, but without any formal validation using Jersey data, this is an excessive claim. I would suggest limiting these claims to Holsteins and discussing the likely limitations in Jerseys, or simply dropping all Jersey results. You wrote, 'Our findings underscore the advantages of using breed-specific pangenome references for SV genotyping and imputation', but a multi-breed pangenome would probably be a better option for multi-breed targets.

AU: We thank the reviewer for this important point. We agree that our current pangenome panel is Holstein-specific, and imputation accuracy is expected to be highest in Holsteins. While some

common SVs may be imputed in Jerseys, breed-specific or rare SVs in Jerseys are unlikely to be accurately captured without validation. To address this, we have revised the text to limit claims to Holsteins and added a discussion of likely limitations in applying the panel to other breeds. Specifically, we now state that on L441: "Our findings underscore the advantages of using breed-specific pangenome references for SV genotyping and imputation in Holsteins. We note that multi-breed pangenomes would likely be more appropriate for imputation across breeds, as they could capture rare and breed-specific SVs not represented in a single-breed reference." This revision clarifies the scope of applicability and acknowledges the need for multi-breed pangenomes for broader use.

Q39. 2. The panel is already quite good but it is still limited to 40 haplotypes. Therefore only SVs present in these 40 haplotypes can be imputed. It would be worth to be discussed.

AU: Indeed, the current pangenome panel includes 40 haplotypes, which limits imputation to SVs represented in these haplotypes. Rare or private SVs not captured in the panel cannot be imputed accurately, which constrains the overall completeness of SV genotyping. On L475, we have added "Expanding the panel with additional haplotypes, particularly from diverse populations or long-read assemblies, would likely improve the ability to impute a broader spectrum of SVs."

Q40. 3. The GWAS thresholds are low (-log(p)=6.1). The SVs are indeed compared with all variants, including SNPs, and the total number of tests to consider is therefore much higher than the number of SVs. Consider, for instance, an SV with -log(p) = 6.5 that is close to a SNP with -log(p) = 10. This SV would not be a relevant candidate variant.

AU: We thank the reviewer for this important point. The GWAS significance threshold for SVs ($-\log_{10}(p) \approx 6.1$) was determined by Bonferroni correction based on the number of SVs tested ($p = 0.05 / 68{,}354 \approx 7.3 \times 10^{-7}$). This threshold is appropriate for the SV-specific GWAS, as it is designed to screen for significant SV-trait associations within the SV variant space, rather than to directly compare or rank SVs against SNPs.

We agree with the reviewer that an SV passing this threshold does not necessarily represent a causal variant, particularly when a nearby SNP shows a stronger association. To address exactly this concern, we performed joint fine-mapping incorporating both SNPs and SVs, using PIP to evaluate the likelihood of each variant being causal. Only 32 SVs with PIP ≥ 0.9 were highlighted as strong candidate causal variants (Table 1). This joint fine-mapping framework effectively accounts for the scenario described by the reviewer, as an SV with modest significance near a much stronger SNP signal would receive a low PIP and would not be prioritized as a candidate causal variant.

We have added clarifying language in the manuscript to emphasize that the SV-GWAS serves as an initial discovery step, while the joint fine-mapping provides the rigorous framework for evaluating causality across variant types.

Q41. 4. Imputation with SNP variants raises the issue of variants being included in deletions. It seems that the authors included all SNPs for imputation of deletions. I have a feeling that some results for deletions could be unpredictable or false. I would strongly recommend excluding these SNPs present in deleted sequences from the SV imputation process.

AU: We appreciate the reviewer's concern regarding the inclusion of SNPs located within deleted regions during SV imputation. Importantly, in the PanGenie-based framework used in this study, SNPs residing within deletion intervals are not genotyped, as the corresponding reference sequence is absent in individuals carrying the deletion. Consequently, these SNPs do not contribute genotypic information for deletion carriers and are not directly used to drive deletion imputation.

Instead, deletion imputation primarily relies on flanking SNPs outside the deleted regions, which remain genotyped and provide LD information for predicting deletion status. Therefore, SNPs physically located within deleted sequences do not introduce spurious signals into the deletion imputation process.

Q42. 5. Could you please characterise the complex variations? I would guess that they comprise both a deletion and an insertion, i.e. they are big 'substitutions'. Could you confirm this? How did you handle insertions with a few bases deleted or deletions with a few bases inserted, which are very common?

AU The COMPLEX variants in our study generally represent SVs that involve more than one type of structural change, most commonly combinations of insertions and deletions, effectively acting as large substitutions.

For small-scale events, such as insertions with a few deleted bases or deletions with a few inserted bases, these were treated according to the predominant variant type. If the net change was primarily an insertion, the variant was classified as an INSERTION; if primarily a deletion, it was classified as a DELETION. Variants with multiple overlapping changes that could not be resolved as a single insertion or deletion were categorized as COMPLEX.

This approach ensures that small "mixed" events are captured within the main SV types, while larger, truly multi-component variants are grouped as COMPLEX. We have clarified this classification on L106.

Q43. 6. The effect of MATL3 on stature has already been published by Sorin et al.: https://doi.org/10.1186/s12711-025-01012-x. Please cite this reference and adjust your text. Did you check the F1/genotype concordance of genotyping SVs from PanGenie with ones called using HiFi data?

AU We have added a citation to the recent study by Application of a French cattle pangenome, from structural variant discovery to association studies on key phenotypes (Sorin et al., 2025) that reports a deletion affecting MATN3 associated with stature in cattle.

Regarding concordance between SVs imputed by PanGenie and SVs called from high-fidelity (HiFi) long-read data: in our study we did not perform a formal cross-validation by comparing every imputed SV genotype with matched long-read SV calls. Our design focused on imputation performance metrics (F1, precision, sensitivity) based on internal cross-validation rather than HiFi validation. We acknowledge this as a limitation. We now discuss this in the revised manuscript and note that future work using matched long-read sequencing data will be valuable to assess the true accuracy and concordance of imputed SV genotypes against a high-quality long-read ground truth.

Q44. 7. Perhaps we could include a discussion about the computational resources required to genotype the SVs from 1,000 animals using PanGenie?

AU: We thank the reviewer for suggesting the inclusion of computational resource requirements. We have added a discussion on the computational cost of large-scale SV genotyping using PanGenie.

In our experience, genotyping approximately ~43,000 SVs per chromosome from whole-genome sequencing data at ~10× coverage can be performed using a single CPU core with ~16 GB of memory, with a wall-clock time of approximately 4–8 hours per sample, depending on the number of SVs, sequencing depth, and sample complexity. When applied to ~1,000 animals on a high-performance computing cluster, the total runtime is efficiently reduced through straightforward sample- and chromosome-level parallelization.

Importantly, the computational requirements of PanGenie are modest compared to assembly-based or long-read-based SV discovery approaches, which typically require substantially greater CPU time, memory, and storage. Runtime scales approximately linearly with the number of samples and SVs, making PanGenie a practical and scalable solution for population-scale SV genotyping.

Other remarks:

Q45. 137: Fix typo.

AU: We have corrected the typo.

Q46. 214–16: You state that the results for Jersey are better than those for hybrids. This is not logical given the nature of the reference panel. Is this due to the higher number of homozygous SVs?

AU: We agree that, at first glance, it may seem unexpected for Jersey to show better imputation performance than hybrid animals when using a Holstein-based reference panel. However, this pattern is biologically and methodologically explainable.

Because the reference pangenome is constructed from Holstein genomes, many Jersey-specific SVs are not represented in the reference panel and are therefore excluded from the evaluation. The SVs that remain shared between Holstein and Jersey are predominantly ancestral or common variants, which tend to occur at higher frequencies and exhibit a higher proportion of homozygous genotypes in Jersey. Homozygous SVs are intrinsically easier to impute than heterozygous ones due to clearer genotyping signals and reduced phasing ambiguity.

In contrast, hybrid populations contain a larger fraction of heterozygous and admixed SV genotypes, which are more difficult to impute accurately because of increased haplotype complexity and weaker LD with the reference panel. Consequently, the observed higher imputation accuracy in Jersey compared to hybrids is an expected outcome given the Holstein-based reference and the underlying genotype composition.

Q47. 238, 585: Please clarify the number of SNPs for RNA. You mentioned 1,451,919, but it seems that only a small proportion were kept.

AU: Only the subset of SNPs retained after quality control filtering was used for imputation. The initial RNA-seq variant set was filtered to remove low-quality and non-informative variants, resulting in a final set of 1,451,919 RNA SNPs used for downstream analyses. We have clarified both the filtering process and the final number of SNPs used in the Methods (Lines 281).

Q48. 245–246: Can you explain why including indels decreases accuracy?

AU: On L256, we added “likely because indels are more difficult to genotype reliably and can disrupt haplotype phasing or marker consistency, thereby introducing additional noise into the imputation process.”

Q49. 257: Replace "an MAF" with "a MAF".
AU: Done.

Q50. 302: Remove 'successful' as there was no validation of the Jersey results.
AU: Removed.

Q51. 309: Net merit is a combination of many traits. I suggest removing this trait.
AU: We thank the reviewer for the comment. Net merit is indeed a composite trait, but it remains a key selection index widely used in the U.S. dairy industry. We have therefore retained it in the analysis to ensure our findings remain directly relevant for practical dairy breeding applications, while noting that it combines multiple correlated traits.

Q52. 318: Fix the typo in functions.
AU: Done.

Q53. 678: Milk yield and fat yield are not uncorrelated; the correlation is around 0.6 or 0.7. It would be better to say that highly correlated traits were avoided.
AU: On L647, we have modified the text “we tried to avoid highly correlated traits within each group based on genetic correlation estimates”.

Q54. 567: I do not understand Fst in '(1 FST 0%, 20%, and 50%)'.
AU: We apologize for the typo. The text “1 FST” was unintended and has been removed. The corrected sentence now reads: “We randomly introduced missing values at different rates (10%, 20%, and 50%) for each sample.”

Q55. 613: Please provide the minimum and mean (or median) number of daughters per bull.
AU: We have provided the minimum and mean number of daughters per bull for the milk traits. On L671, we added “For example, for milk fat, the minimum and mean numbers of daughters per bull are 37 and 2,764 in the 173-bull dataset, and 10 and 1,106 in the 52,099-bull dataset.”

Q56. 667: How did you treat weights when R^2 was close to 1? Did you bound reliability to avoid very high weights?
AU: We thank the reviewer for the comment. In our formula, **R** is the residual variance scaling. Under our parameterization, $R_{ii} = 1/r_i^2 - 1$, where r_i^2 is the reliability of the *i*-th individual's dPTA, so when reliability r_i^2 is close to 1, R_{ii} becomes small. Individuals with higher reliability receive smaller residual variance, which is the expected behavior for more accurately estimated breeding values. No reliability values were exactly 1.

** See Nature Portfolio’s author and referees' website at www.nature.com/authors for information about policies, services and author benefits.

This email has been sent through the Springer Nature Tracking System NY-610A-NPG&MTS

Confidentiality Statement:

This e-mail is confidential and subject to copyright. Any unauthorised use or disclosure of its contents is prohibited. If you have received this email in error please notify our Manuscript Tracking System Helpdesk team at http://platformsupport.nature.com . Details of the confidentiality and pre-publicity policy may be found here http://www.nature.com/authors/policies/confidentiality.html

Privacy Policy | Update Profile

Thank you again for submitting your revised manuscript "An enhanced pangenome reference panel enables accurate imputation of structural variation and large-scale genotype-phenotype analyses in dairy cattle" to Nature Communications. We have now received reports from the reviewers who evaluated the original version. On the basis of their comments (copied below), we have decided to invite an additional revision of your work.

Q1. You will see that, while the reviewers find that your revisions improved the manuscript, some important points remain to be addressed. Specifically, you will have to provide a Github repository with the scripts needed to reproduce your results, since it will not be sufficient to state that this will be done. Additionally, please ensure that you address Reviewer #3's remaining points. I strongly suggest removing the Jersey results or moving them to the Supplementary. Please revise your manuscript, addressing all the remaining issues raised by the reviewers.
AU: Please see our answers to Q 2 and Q3 below.

…
REVIEWER COMMENTS

Reviewer #1 (Remarks to the Author):

The responses to reviewers critiques were acceptable in most cases.

Reviewer #2 (Remarks to the Author):

Q2. Authors addressed all my concerns, except the reproducibility one. They describe a promise to create a detailed GitHub repository, which would be very appreciated, but it is something that I cannot review because it does not yet exist. Authors should make the GitHub repository available before acceptance.
AU: We have now made the GitHub repository publicly available at https://github.com/xyxss/cattlePanSVimp and https://github.com/JJWang259/sv-association-pipeline. It includes all necessary scripts for data preprocessing, SV genotyping using PanGenie, imputation with Beagle, and the downstream GWAS/fine-mapping analyses (https://github.com/jiang18/gemrich) to ensure full reproducibility.

Reviewer #3 (Remarks to the Author):

The manuscript has undergone significant revision, for which I thank the authors. However, I still have some remarks that need to be addressed:

Q3. Regarding the imputation of Jersey, you have only added a few lines of discussion but have maintained most of your results. I am not at all confident about the Jersey results because we know that it does not work well with SNPs. Only frequent SV shared across breeds can be imputed. If you really want to maintain these results, you should mention from the beginning that it is a (unvalidated) test.

AU: We thank the reviewer for raising this important point regarding cross-breed imputation. We acknowledge that previous research, particularly trials using SNP-based methods in various European populations, has encountered significant challenges in this area. However, we believe our findings provide a useful perspective on the potential for SV imputation when using a pangenome framework. As the reviewer correctly notes, imputation accuracy is generally more reliable for frequent SVs shared across breeds. Our results are likely to reflect the successful capturing of this shared ancestral variation, facilitated by the high-density SV catalog and the pangenome reference. We recognize that imputation performance is contingent on a complex interplay of factors, including the quality of the reference, variant frequency and density, and the genetic distance of the involved breeds. To ensure a balanced and transparent presentation, we have retained these empirical results in the main text but have carefully revised the manuscript to describe this specific analysis as an **'exploratory and unvalidated test.'** We have also added caveats (e.g., Lines 230 and 504) emphasizing that these results should be interpreted with caution, particularly for low-frequency or breed-specific variants. We hope this added framing addresses the reviewer's concern while providing valuable context for future cross-breed studies.

On L230, we modified to "Contrary to expectations, Jersey samples did not show lower imputation accuracy with the Holstein pangenome reference (**Figure 2c**). **We consider this a preliminary and unvalidated test of cross-breed imputation**. The high performance observed is likely driven by a high proportion of frequent, homozygous SVs in Jerseys that are shared with the Holstein reference. However, we caution that accuracy for rare or breed-specific variants is expected to be significantly lower."

On L509, we modified to "We note that while multi-breed pangenomes would be ideal for cross-breed applications, our Holstein-specific panel performed surprisingly well on Jersey samples. This exploratory test suggests that shared common SVs can be accurately captured across breeds, though we emphasize that these results depend on variant density and allele frequency, and should be viewed **as unvalidated for general cross-breed use**."

Q4. Lines 510-515, you stated in your rebuttal letter that Sorin et al. (https://doi.org/10.1186/s12711-025-01012-x), who demonstrated the impact of MATN3 deletion on stature, are now cited. However, I cannot find this reference in the manuscript. Please add this reference.

AU: We apologize for this oversight. We have now added the reference: Sorin, et al. (2025). Genet Sel Evol 57(1):61 to the manuscript at L515 where the MATN3 deletion is discussed.

Q5. Regarding the use of SNPs located in a deletion, I am not at all convinced by your answer. Of course, for SNPs located in deleted sequences, genotypes are missing for homozygous deleted individuals and there is only one allele for heterozygous individuals. It is very likely that they are imputed incorrectly in the imputation process and this can provide unpredictable results. I acknowledge that removing them would have implied to recompute all imputations and GWAS from scratch, but at least discuss that point in the manuscript.

AU: Thank you for this insight. We agree that SNPs within deleted regions are a

significant concern for imputation accuracy. We would like to clarify that our pipeline inherently avoids this issue: because PanGenie was used for genotyping, SNPs located within regions identified as deletions are not reported and were therefore never included in the imputation reference panel. We have added a paragraph to the Discussion (Lines 506–510) clarifying this: Crucially, our k-mer–based genotyping approach via PanGenie effectively addresses a common challenge in SV imputation: the presence of SNPs within deleted sequences. Such SNPs typically lead to inaccurate results due to missing genotypes in homozygous deletion carriers and reduced allelic information in heterozygotes. In our pipeline, these variants are inherently excluded from the reference panel, ensuring that the resulting imputation is not confounded by “phantom” SNPs in deleted regions and avoiding potential biases in downstream analyses.

Q6. Abstract: You mention 1,225 SV-trait associations, but it would be more accurate to state that only 32 of these are good candidate variants and the rest are merely associations.
AU: We have revised the Abstract (L46) to distinguish between the total number of associations and fine-mapped candidates. It now reads: '...identified 1,225 SV-trait associations, of which 32 high-confidence candidate SVs were prioritized through fine-mapping, including a 75-bp deletion in *ANKRD11*...'"

Q7. Line 473, the author wrote: 'Consistent with long homozygous haplotypes shaped by intensive selection.' A more likely explanation is that the minor allele frequency (MAF) is quite low, and homozygous variants (mainly with the major allele), are much easier to impute than other genotypes.
AU: We agree with the reviewer that the low MAF of many variants contributes significantly to high imputation accuracy for homozygous major-allele genotypes. We have revised the text (Line 473) to acknowledge that while selection shapes these haplotypes, the high accuracy is also a reflection of the allele frequency spectrum and the relative ease of imputing common major-allele genotypes. On L476, we modified it to “A notable finding of our study is the superior imputation accuracy for homozygous SV genotypes (F1 = 0.93) compared to heterozygous ones (F1 = 0.84). This result likely stems from a combination of biological factors, such as selection-driven homozygous haplotypes, and technical factors, specifically **the increased reliability of imputing common major alleles across the frequency spectrum**.”

Q8. 463: Fix typo in review.
AU: Done
